# The Rho GEF Trio functions in contact inhibition of locomotion of neural crest cells by interacting with Ptk7

**DOI:** 10.1242/dev.204446

**Published:** 2025-05-06

**Authors:** Katharina Till, Annette Borchers

**Affiliations:** Department of Biology, Molecular Embryology, Philipps-University Marburg, Karl-von-Frisch-Straße 8, 35043 Marburg, Germany

**Keywords:** Neural crest migration, RhoGEF, Cell-cell contacts, Contact inhibition of locomotion

## Abstract

Neural crest (NC) cells are highly migratory cells that contribute to a wide range of vertebrate tissues and must respond to a variety of external signals to precisely control directed cell migration. The RhoGEF Trio is particularly well suited to relay signals to the cytoskeleton because it contains two GEF domains that activate Rac1 and RhoA, respectively. Previously, we have shown that Trio is dynamically localized in *Xenopus* NC cells and required for their migration. However, how its distinct enzymatic functions are spatially controlled remains unclear. Here, we show that Trio is required for contact inhibition of locomotion (CIL), a phenomenon whereby NC cells change their polarity and directionality upon cell-cell contact. At cell-cell contacts, Trio interacts with Ptk7, a regulator of planar cell polarity that we have recently shown to be required for CIL. Our data suggest that Ptk7 inhibits the Rac1 activity of Trio, thereby limiting Trio activity to the activation of RhoA and promoting CIL.

## Teaser

**Summary:** Migrating neural crest cells use Ptk7 to locally restrict the activity of the RhoGEF Trio to the activation of RhoA at cell-cell contact sites.

## INTRODUCTION

Cell migration is important for embryonic development, but also for the maintenance of adult organisms by controlling important processes such as wound healing or the immune defense. Neural crest (NC) cells provide an ideal model for studying the principles of cell migration, as they migrate over long distances throughout the embryo to give rise to a variety of derivatives, including neurons and glia cells, cartilage, bone and pigment cells. Collective migration of NC cells is controlled by a combination of factors, including chemoattractants, guidance cues, a confinement effect of the surrounding tissue and dynamic NC cell-cell interactions, such as contact inhibition of locomotion (CIL) (Szabo and Mayor, 2018). CIL describes a cell behavior whereby cells change their polarity and direction of migration after cell-cell contact (Roycroft and Mayor, 2016). In NC cells, CIL contributes to the dispersion and directional migration of NC cells (Carmona-Fontaine et al., 2008; Theveneau et al., 2010). Upon cell-cell contact, cell-cell adhesion complexes form (Becker et al., 2013; Scarpa et al., 2015; Theveneau et al., 2010), planar cell polarity (PCP) Wnt signaling is activated, including the recruitment of the Frizzled receptor Fz7 and Dishevelled (Dvl), leading to activation of the small GTPase RhoA (Carmona-Fontaine et al., 2008). Conversely, the small GTPase Rac1 is excluded from the contact area, leading to an inhibition of protrusive activity in the direction of the contact. At cell-cell contacts, cell protrusions collapse and Rac1 is activated at the free edge facing away from the cell contact, where it contributes to the formation of new protrusions (Szabo and Mayor, 2018). Thus, migrating NC cells must respond very dynamically to incoming signals and/or cues, and relay them efficiently to fine-tune directed migration.

The RhoGEF TRIO is ideally suited to transmit signals to regulate cell migration, as it contains three functional domains: two GEF domains and a serine/threonine kinase domain (Debant et al., 1996). TRIO controls the activity of Rho GTPases by activating Rac1 and RhoG, via its N-terminal GEF1 domain, or RhoA, via its C-terminal GEF2 domain (Bellanger et al., 1998; Blangy et al., 2000; Debant et al., 1996). That TRIO is involved in the regulation of cell migration is also reflected by its choice of interaction partners, which include actin regulators such as Filamin A, CARMIL and TARA (Bellanger et al., 2000; Vanderzalm et al., 2009; Yano et al., 2011), cell adhesion molecules like members of the cadherin family, or mediators of cell-matrix interactions such as focal adhesion kinase (FAK) (Backer et al., 2007; Charrasse et al., 2007; Kashef et al., 2009; Medley et al., 2003; Timmerman et al., 2015). Consistently, TRIO expression is deregulated in several types of human cancers (Adamowicz et al., 2006; Feng et al., 2014; Lane et al., 2008; Salhia et al., 2008; Wang et al., 2015; Yoshizuka et al., 2004; Zheng et al., 2004), further supporting its crucial role in cell migration. In addition, TRIO mutations have been identified in individuals with neurodevelopmental disorders and variable craniofacial anomalies (Ba et al., 2016; Barbosa et al., 2020; Gazdagh et al., 2023; Katrancha et al., 2017; Pengelly et al., 2016; Sadybekov et al., 2017). Recently, two mutation hotspots, one in the spectrin repeat domain and one in the GEFD1 domain, have been identified that lead to hyper- and hypoactivation of Rac1, respectively, with Rac1 levels correlating with the head size in patients (Barbosa et al., 2020). Mechanistically, the spectrin repeat domain of TRIO binds to the pleckstrin homology region of the GEF1 domain and inhibits its binding to Rac1 (Bircher et al., 2022). Factors such as the cell-adhesion molecule VE-cadherin, which binds to the spectrin repeat region of zebrafish Trio (Timmerman et al., 2015), can release this autoinhibition, thereby preventing its interaction with the GEF1 domain and leading to hyperactivation of Rac1. Thus, the unique structure of TRIO may allow the local fine-tuning of its catalytic activities. However, how this is controlled during cell migration remains unclear.

During *Xenopus* development, Trio is required for NC cell migration and cartilage formation, where it functions cell-autonomously upstream of Rac1 and RhoA (Kratzer et al., 2020). The Trio loss-of-function defects can be rescued by expression of the Trio GEF2 domain. Since we have shown that the GEF2 domain interacts with Dvl and, furthermore, that Dvl can rescue Rac1 activity in Trio morphant embryos, this suggests that Trio controls NC migration by interacting with Dvl to activate Rac1 (Kratzer et al., 2020). Interestingly, the NC migration defects observed in Trio morphant NC cells are identical to those observed in Ptk7 morphants (Podleschny et al., 2015). Ptk7, originally identified as colon carcinoma kinase 4 (CCK4) (Mossie et al., 1995), is a member of the receptor tyrosine kinase family with extracellular immunoglobulin-like domains and an evolutionary conserved inactive tyrosine kinase domain (Miller and Steele, 2000; Sheetz et al., 2020). In vertebrates, Ptk7 plays a role in the regulation of PCP (Andreeva et al., 2014; Hayes et al., 2013; Lee et al., 2012; Lu et al., 2004; Paudyal et al., 2010; Williams et al., 2014; Xu et al., 2016; Yen et al., 2009) and functions as a Wnt co-receptor (Bin-Nun et al., 2014; Martinez et al., 2015; Peradziryi et al., 2011; Podleschny et al., 2015). Like Trio, Ptk7 is required for NC migration and cartilage formation (Shnitsar and Borchers, 2008). Recently, we have shown that Ptk7 is dynamically localized at NC cell-cell contact zones, where it plays a role in CIL (Grund et al., 2021). Using deletion constructs, we showed that the extracellular immunoglobulin-like domains of Ptk7 mediate homophilic binding and are relevant for the transient accumulation of Ptk7 at cell-cell contact zones. Loss of Ptk7 function abolished CIL, and morphant NC explants were invaded by wild-type NC cells (Grund et al., 2021). Since Ptk7 and Trio morphants show these remarkable similarities, we investigated here whether these molecules also interact functionally. Our data suggest that interaction of Trio with Ptk7 contributes to the fine-tuning of Trio function at cell-cell contacts.

## RESULTS

### Trio rescues the Ptk7 morphant NC phenotype

**Fig. 1.**
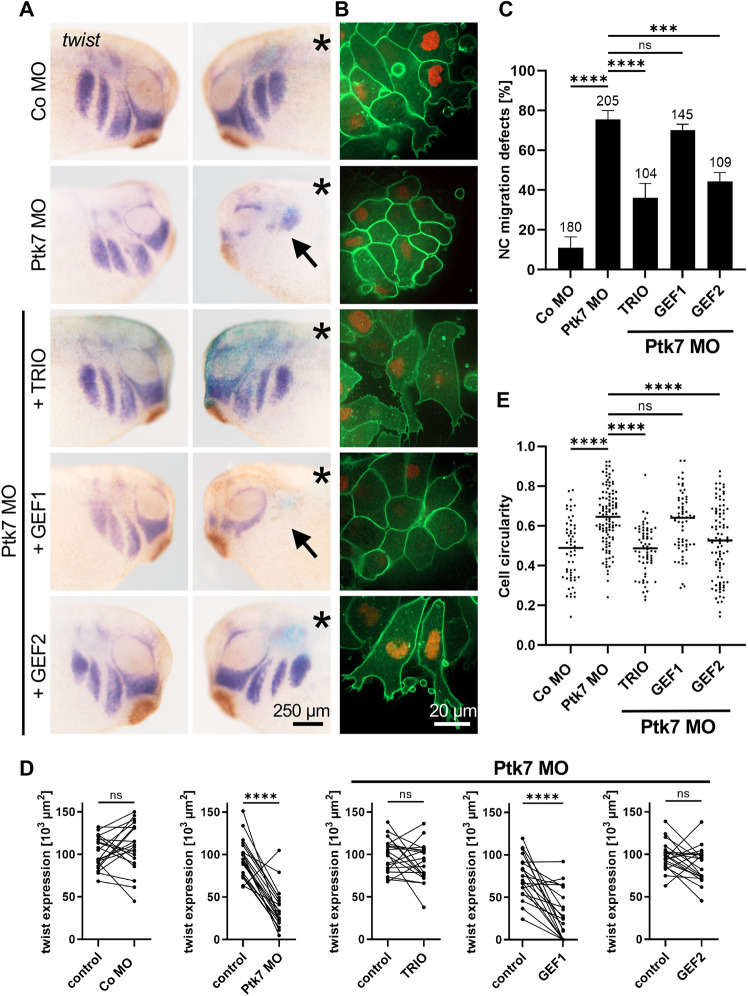
**The Trio GEF2 domain rescues the Ptk7 morphant NC migration defect.** (A,B) Two-cell stage embryos were injected with 6.5-7.5 ng Ptk7 MO or a control MO in combination with 75 pg *lacZ* RNA (A) or 50 pg *mbGFP* RNA together with 250 pg *H2B-mcherry* RNA (B) as lineage tracer. Co-injection of 150 pg TRIO DNA and 100 pg *GEF2* RNA, but not 100 pg *GEF1* RNA, restored NC cell migration *in vivo* and protrusion formation *in vitro* of Ptk7 morphants. (A) NC cell migration was analyzed at stage 26 by *twist in situ* hybridization. Asterisks mark the injected side. Arrows indicate NC migration defects. Scale bar: 250 µm. (B) NC cells were explanted at stage 18, cultured on fibronectin-coated slides and analyzed by time-lapse microscopy. Scale bar: 20 µm. (C) Graph summarizing the percentage of embryos with NC migration defects of at least three independent experiments. The total number of embryos is indicated for each column. Data are mean±s.e.m. ****P<0.0001, ***P<0.001; ns, not significant (one-way ANOVA). (D) Quantification of the *twist in situ* hybridization shown in A. Graphs presenting the measured area of *twist* expression of 20 randomly selected embryos per condition. The three graphs on the right depict the results of embryos co-injected with Ptk7 MO and the respective rescue constructs. The *twist* expression of the uninjected side (control) is compared to that of the injected side of the embryo. ****P<0.0001; ns, not significant (paired *t*-test). (E) A graph plotting cell circularity of explanted NC cells shown in B. Number (*n*) of analyzed cells: Co MO=57, Ptk7 MO=111, Ptk7 MO+TRIO=66, Ptk7 MO+GEF1=63 and Ptk7 MO+GEF2=92. The median is plotted as a line. ****P<0.0001; ns, not significant (Kruskal–Wallis test).

Since Trio and Ptk7 loss of function caused a strikingly similar phenotype in *Xenopus laevis* NC development, we tested whether they also interact functionally. To this end, embryos were injected in one blastomere at the two-cell stage with control morpholino (Co MO) or Ptk7 morpholino (Ptk7 MO) in combination with different Trio constructs. For the analysis of NC migration in whole embryos, embryos were co-injected with *lacZ* RNA as a lineage tracer (Fig. 1A). To analyze the migration of explanted NC cells, embryos were co-injected with *mbGFP* RNA and *H2B-mCherry* RNA to label the membrane and nucleus, respectively (Fig. 1B). *In vivo* NC migration was analyzed in neurula and tailbud stage embryos using *in situ* hybridization for the NC marker *twist* (*twist1*) (Fig. 1A, Fig. S1). Although no significant defects were observed at stages of NC induction (Fig. S1), Ptk7 morphant embryos showed significant defects in NC migration compared to controls (Fig. 1C). These were also apparent when the *twist* expression area in the branchial arches between the injected and non-injected side were compared (Fig. 1D). Ptk7 morphants showed a significant decrease in the *twist* expression on the injected side compared to the uninjected side, while no significant defects were observed in control MO-injected embryos. As previously observed (Podleschny et al., 2015), time-lapse analysis of explanted cranial NC cells showed that, in contrast to control NC cells, Ptk7 morphant cells lost the ability to form filopodia and lamellipodia, and instead exhibited membrane blebbing (Fig. 1B). These findings were confirmed by a significant increase in cell circularity of Ptk7 morphant cells compared to controls (Fig. 1E). Interestingly, co-expression of full-length human TRIO DNA restored *in vivo* cranial NC migration of Ptk7 morphants (Fig. 1A,C). Furthermore, protrusion formation of Ptk7 morphant NC explants was also rescued by co-expression of TRIO (Fig. 1B,E). Thus, Trio likely functions downstream of the Ptk7 receptor in NC migration.

To test whether the GEF domains of Trio are functionally important in this process, we performed rescue experiments with *Xenopus* Trio-GEF1 or Trio-GEF2 constructs, consisting of the respective DH, PH and SH3-domain. Notably, co-expression of the GEF2 domain, but not the GEF1 domain, was sufficient to rescue NC migration in Ptk7 morphant embryos (Fig. 1A,C). This result was also confirmed by comparing the *twist* expression area between the injected and non-injected side of the embryo: co-injection of the GEF2 domain restored *twist* expression on the Ptk7 MO-injected side compared to the uninjected side, while co-expression of the GEF1 domain did not (Fig. 1D). This effect was also observed at the cellular level, where only the GEF2 domain restored protrusion formation, resulting in a reduction in cell circularity, while the GEF1 domain failed to rescue the cell migration defects (Fig. 1B,E). It is known that the activity of the GEF1 domain is controlled by an intramolecular folding of the N-terminal spectrin repeat domain to the pleckstrin homology region of the GEF1 domain, thereby sterically preventing its binding to Rac1 (Bircher et al., 2022; Bonnet et al., 2023). Thus, one technical explanation for the inability of the GEF1 domain to rescue NC cell migration in Ptk7 morphants may be the lack of its adjacent regulatory domain, possibly leading to hyperactivation of Rac1. However, a construct containing the GEF1 domain and the complete N-terminal segment of TRIO, including the spectrin repeat domain (TRIO-N1) (van Haren et al., 2014), was also not sufficient to restore NC migration in Ptk7 morphant embryos (Fig. S2). Taken together, these results suggest that Trio functions downstream of Ptk7 during NC cell migration and that the GEF2 domain is crucial in this process.

### TRIO interacts with Ptk7 in NC cells

**Fig. 2.**
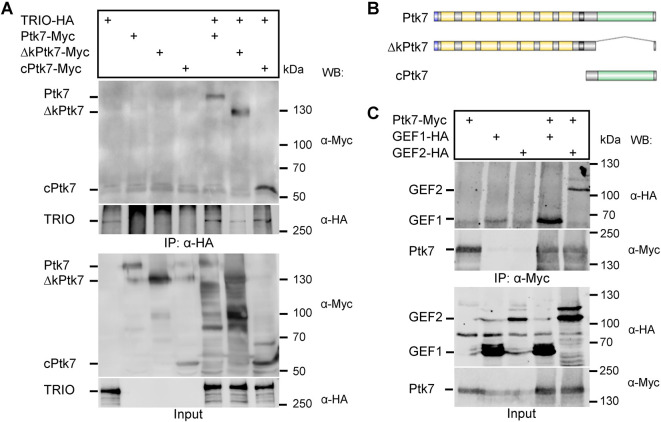
**TRIO interacts with Ptk7 in HEK293 cells**. (A-C) HEK293 cells were transfected as indicated and immunoprecipitations were performed using anti-HA (A) or anti-Myc (C) antibodies. Ptk7 constructs used for immunoprecipitation are shown in B. (A,C) Co-immunoprecipitated proteins are shown in the upper panel, immunoprecipitated proteins in the middle panel and cell lysates in the bottom panel. Antibodies used for western blotting and molecular weights (kDa) are indicated on the right. Representative results of at least three independent experiments are shown.

Since Trio and Ptk7 appear to be involved in the same pathway during NC cell migration, we performed co-immunoprecipitation (co-IP) to analyze whether the proteins might interact with each other. Myc-tagged Ptk7, a kinase domain deletion mutant (ΔkPtk7), and a construct consisting of only the cytoplasmic region of Ptk7 (cPtk7) were co-transfected with TRIO-HA and co-IPs were performed using anti-HA antibodies (Fig. 2A,B). TRIO co-precipitated all tested Ptk7 constructs. The only common region of the deletion constructs is the intracellular domain adjacent to the transmembrane domain, suggesting that this juxtamembrane domain is likely required for the interaction of Ptk7 and TRIO. To analyze the role of the Trio-GEF domains in this interaction, HEK293 cells were co-transfected with HA-tagged GEF1 or GEF2, together with Ptk7-Myc. Interestingly, Ptk7 co-precipitated both the GEF1 and GEF2 domains, and vice versa (Fig. 2B and Fig. S3).

**Fig. 3.**
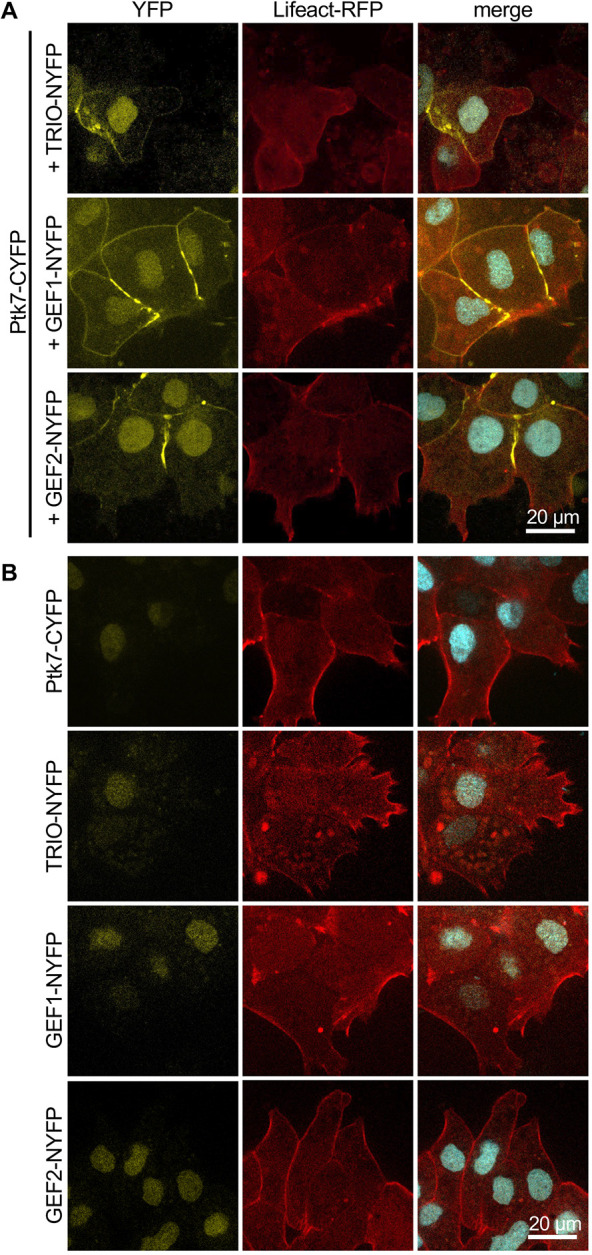
**TRIO interacts with Ptk7 in NC cells.** (A) NC explants were injected with 100 pg *Ptk7-CYFP* RNA in combination with 100 pg TRIO-NYFP DNA, 100 pg *GEF1-NYFP* RNA or 100 pg *GEF2-NYFP* RNA together with 200 pg *H2B-CFP* RNA and 300 pg *lifeact-RFP* RNA in one blastomere at the two-cell stage. Representative results of at least three independent experiments are shown. YFP signal indicates an interaction between Ptk7 and TRIO at NC cell-cell contacts. YFP and CFP signals cannot be distinguished by the imaging system, so nuclear labeling (H2B-CFP) is also visible in the YFP channel. Scale bar: 20 µm. (B) Negative control for the bimolecular fluorescence complementation assay. NC explants were injected with the indicated constructs in combination with *H2B-CFP* RNA and *lifeact-RFP* RNA. No YFP signal was detected at NC cell-cell contacts when the NYFP and CYFP constructs were expressed alone. Scale bar: 20 µm.

Next, we performed a bimolecular fluorescence complementation assay to determine the localization of the Ptk7-TRIO interaction in migrating NC cells. To do so, *Ptk7-CYFP* RNA was co-injected with TRIO-NYFP DNA, *GEF1-NYFP* RNA and *GEF2-NYFP* RNA into one blastomere of two-cell stage embryos. *Lifeact-RFP* RNA and *H2B-CFP* RNA were co-injected to label the actin cytoskeleton and the nucleus, respectively. NC cells were explanted at stage 18, cultivated on a fibronectin-coated chamber slide and analyzed using spinning disk microscopy. A YFP signal is detected when a protein marked by a C-terminal YFP comes in close proximity to a protein marked with a N-terminal YFP. Consistent with the co-IP results, a YFP signal was observed for all conditions, indicating that Ptk7 interacts with TRIO, GEF1 and GEF2 in *Xenopus* NC cells (Fig. 3A). The interaction of Ptk7 and TRIO was detected at NC cell-cell contacts. In contrast, no YFP signal was observed at cell-cell contacts when the constructs were expressed alone (Fig. 3B) or when Ptk7-CYFP was co-expressed with NYFP (Fig. S4). Taken together, these data support the hypothesis that Trio is part of a Ptk7 complex localized at cell-cell contacts of migrating NC cells.

### Trio is required for CIL but is not relevant for the protection of NC cells from NC cell invasion

**Fig. 4.**
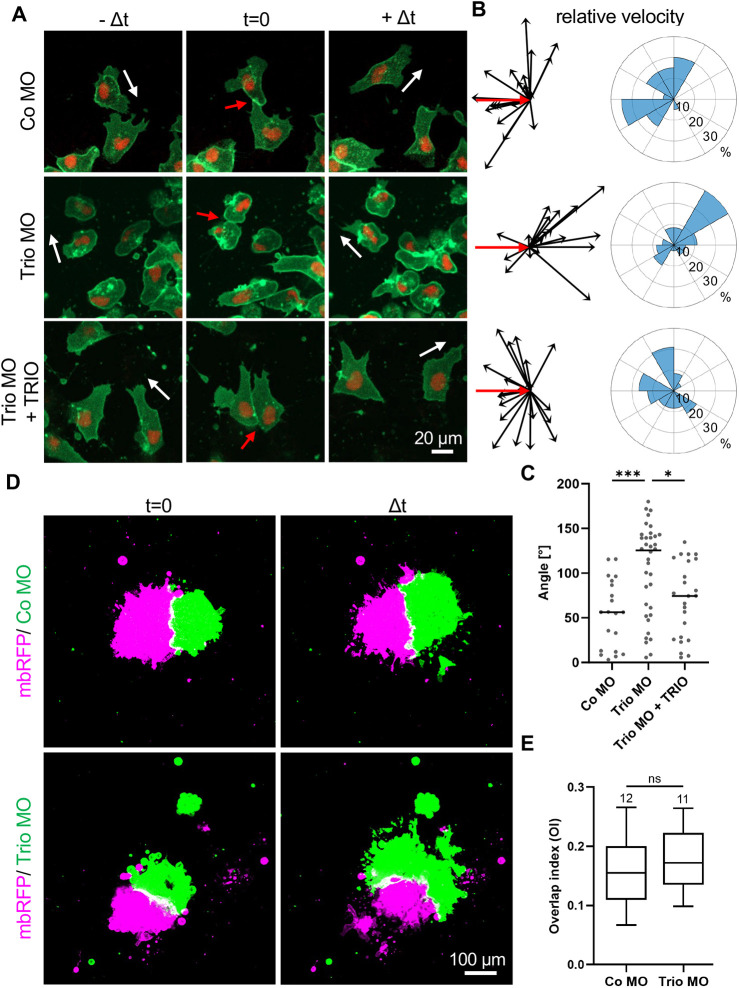
**Trio is required for contact inhibition of locomotion.** (A-C) Embryos were injected with 5-6 ng Trio MO or control MO together with 100 pg *mbGFP* RNA and 200 pg *H2B-mCherry* in one blastomere at the two-cell stage. For rescue experiments, 150 pg TRIO DNA was co-injected. NC cells were explanted at stage 18 and imaged after 4 h of cultivation, when the NC cells had started to migrate as single cells. (A) Single NC cells before (−Δt), during (t=0) and after (+Δt) collision. Scale bar: 20 µm. White arrows indicate the direction of migration. Red arrows indicate cell-cell collisions. (B) Relative velocity vectors (black) with initial velocity vector (red) of 20 representative tracked NC cells are shown (left). Rose plots (right) showing the relative velocity vector distribution of all tracked NC cells. Number (*n*) of analyzed NC cells: Co MO=20, Trio MO=36, Trio MO+TRIO=24. (C) Graph presenting the angles between the velocity vectors and the initial vector. Velocity vectors below the initial vector have been horizontally flipped so that all angles are between 0° and 180° for comparison. *P<0.05, ***P<0.001 (Kruskal–Wallis test). (D,E) For confrontation assay, embryos were injected with either 100 pg *mbRFP* RNA alone or 50 pg *mbGFP* together with 4 ng MO in one blastomere at the eight-cell stage. Explanted NC cells were co-cultured and analyzed by time lapse microscopy. (D) Confronted explants at the start of the experiment (time point t=0, left). Confronted explants at the time point of maximum invasion (Δt, right). Scale bar: 100 µm. (E) Box plot showing the overlap index (OI) at the time point of maximal invasion (Δ*t*) for three independent experiments. The number of analyzed confrontation events is indicated for each column. The box extends from the 25th to the 75th percentile, with whiskers indicating the maximum 1.5×IQR. The median is plotted as a line inside the box. ns, not significant (*P*>0.05; Mann–Whitney test).

Since we have recently shown that Ptk7 is required for CIL in NC cells (Grund et al., 2021), and since Trio has been linked to microtubule stability, which is relevant for CIL (Gossen et al., 2024; Moore et al., 2013), single NC cell collision assays were used in this study to analyze whether Trio is indeed required for CIL. Embryos were injected in one blastomere at the two-cell stage with morpholino oligonucleotides in combination with *mbGFP* RNA and *H2B-mCherry* RNA to label the membrane and nucleus, respectively. For rescue experiments, human TRIO DNA, which is not targeted by the Trio MO, was co-injected. NC cells were explanted at stage 18, cultured on fibronectin-coated chamber slides and imaged after ∼4 h when they started to migrate as single cells. NC cells were imaged and tracked both before (−Δt), during (t=0) and after (+Δt) collision (Fig. 4A), resulting in relative velocity vectors with the initial vector shown in red (Fig. 4B). Velocity vectors of 20 representative tracked NC cells and rose plots summarizing the distribution of all colliding NC cells are shown in Fig. 4B (left and right, respectively). For statistical analysis, angles between the velocity vectors and the initial vector were calculated and adjusted to the range of 0° to 180°, with 180° indicating no change in direction (Fig. 4C). Control NC cells displayed the typical CIL response by changing the migration direction upon contact (Fig. 4A,B). However, loss of Trio function resulted in reduced repulsion, as most cells continued to move in the original direction. This effect was rescued by co-expression of TRIO. These findings were confirmed by a significant increase in the angle between the velocity vectors and the initial vector compared to control cells (Fig. 4C). These data indicate that Trio is indeed required for CIL.

To analyze the effect of Trio on cell invasion, we additionally performed a confrontation assay. Therefore, embryos were either injected with Co MO or Trio MO, together with *mbGFP* RNA or with only *mbRFP* RNA in one blastomere at the two-cell stage. NC cells were explanted at stage 18 and MO-injected explants (green) were placed in close proximity to control explants (magenta) on fibronectin-coated chamber slides. Cell invasion was analyzed using spinning disk microscopy. The extent of NC cell invasion was examined by determining the overlap index (OI), as previously described (Becker et al., 2013). NC explants at the start of the experiment (t=0) and at the time point of maximum invasion (Δt) are shown in Fig. 4D. As expected, control cells were not invaded by NC cells, resulting in a low OI (Fig. 4E). Interestingly, NC cells were also not able to invade Trio morphant NC explants, suggesting that the upstream signal/signals relevant for the protection of NC invasion were still intact.

### Activation of RhoA is crucial for Ptk7 downstream signaling

**Fig. 5.**
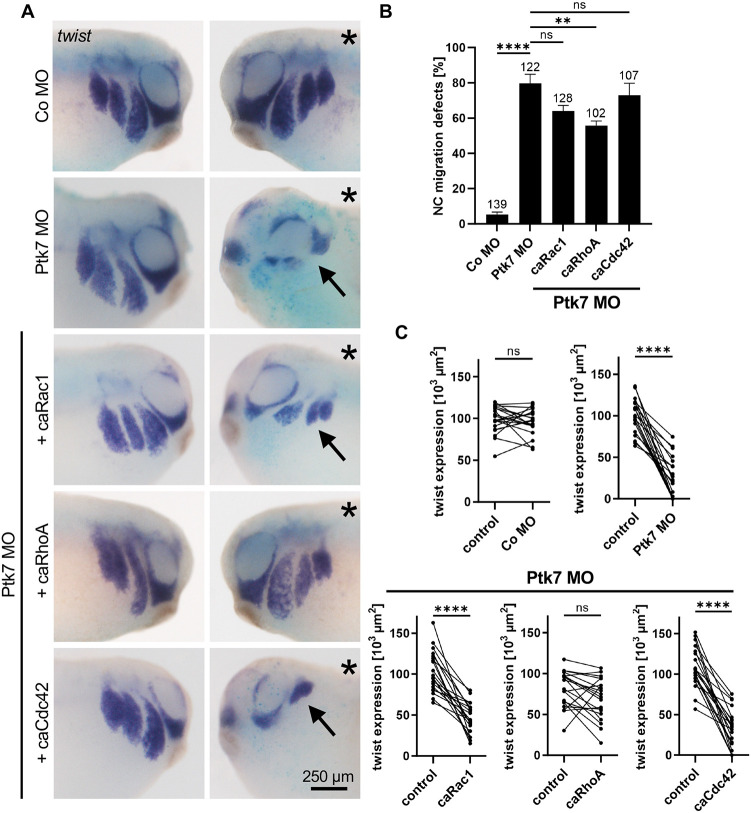
**Activation of RhoA rescues the Ptk7 morphant phenotype.** (A-C) Embryos were injected with 5 ng control MO or 5 ng Ptk7 MO alone or together with 10 pg caRac1, caRhoA or caCdc42 DNA in combination with 50 pg *lacZ* RNA as a lineage tracer in one blastomere at the eight-cell stage. (A) NC cell migration was analyzed at stage 26 by *twist in situ* hybridization. Co-injection of caRhoA rescued NC migration in Ptk7 morphants. The injected side is marked with an asterisk. Arrows indicate NC cell migration defects. Scale bar: 250 µm. (B) Graph showing the percentage of embryos with NC migration defects of at least three independent experiments. The total number of embryos is indicated for each column. Data are mean±s.e.m. ****P<0.0001, **P<0.01; ns, not significant (one-way ANOVA). (C) Graphs presenting the measured area of *twist* expression of 20 randomly chosen embryos per condition. Lower panels show the results of embryos co-injected with Ptk7 MO and the respective rescue constructs. The *twist* expression of the uninjected side (control) is compared to the injected side of the embryo. ****P<0.0001; ns, not significant (paired *t*-test).

Trio has distinct enzymatic functions because it activates Rac1, via its GEF1 domain, and RhoA, via its GEF2 domain. Therefore, we analyzed which of these functions are crucial for its function downstream of Ptk7. To this end, we performed rescue experiments and injected embryos with Co MO or Ptk7 MO in combination with constitutively active (ca) forms of Rac1, RhoA or Cdc42 in one blastomere at the eight-cell stage. *LacZ* RNA was co-injected as lineage tracer and NC cell migration was analyzed in whole embryos using *twist in situ* hybridization (Fig. 5A). Compared to control embryos, Ptk7 MO-injected embryos exhibited severe NC cell migration defects (Fig. 5B). This is also evident in the comparison of the twist expression area between the injected and non-injected side of the embryo (Fig. 5C). Co-injection of caRhoA significantly rescued NC migration in Ptk7 morphant embryos, while caRac1 and caCdc42 did not (Fig. 5A,B). These results are consistent with Trio functioning downstream of Ptk7 with its GEF2 domain, which specifically activates RhoA.

### RhoA activity is decreased in Ptk7 morphant NC cells while Rac1 activity is increased

**Fig. 6.**
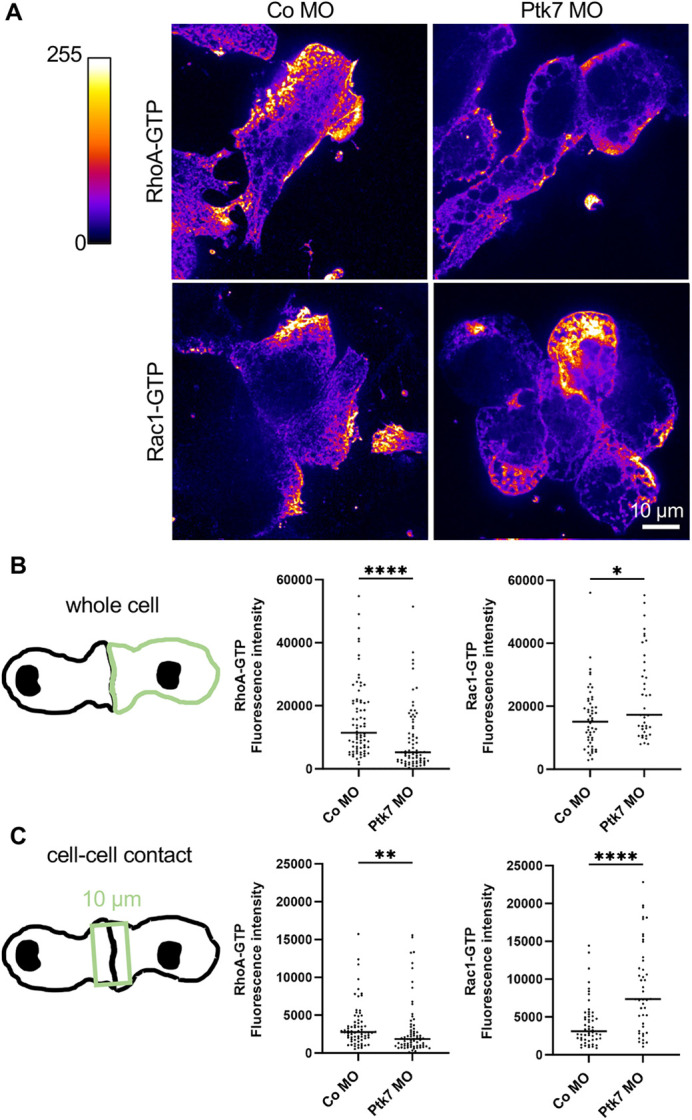
**Loss of Ptk7 function leads to downregulation of RhoA but upregulation of Rac1.** (A) Embryos were injected with 7.5 ng Ptk7 MO or control MO, together with 100 pg *mbGFP* RNA as lineage tracer in one blastomere at the two-cell stage. NC cells were explanted at stage 18 and fixed after 2 h of cultivation. Antibody staining against RhoA-GTP or Rac1-GTP were performed. The ‘Fire’ lookup table of ImageJ was applied. Scale bar: 10 µm. (B) Quantification of the antibody signal of the whole cell. The CTCF is plotted in the graphs. Number (*n*) of analyzed NC cells of at least three independent experiments: RhoA-GTP: Co MO=77, Ptk7 MO=73; Rac1-GTP: Co MO=52, Ptk7 MO=40. *P<0.05, ****P<0.0001 (Mann–Whitney test). (C) Fluorescence intensity at NC cell-cell contact sides. A rectangle with a width of 10 µm was placed over the entire length of the contact (see schematic). Graphs plotting the CTCF of the signal inside the square. Number (*n*) of analyzed cell-cell contacts of at least three independent experiments: RhoA-GTP: Co MO=72, Ptk7 MO=70; Rac1-GTP: Co MO=54, Ptk7 MO=46. **P<0.01, ****P<0.0001 (Mann–Whitney test).

As activation of RhoA can rescue the Ptk7 morphant phenotype, we aimed to analyze whether the activity of RhoA is indeed reduced by Ptk7 loss of function. Therefore, embryos were injected at the two-cell stage with Co MO or Ptk7 MO in combination with *mbGFP* RNA as lineage tracer. NC cells were explanted at stage 18 and immunostained with anti-RhoA-GTP antibodies and, for comparison, with anti-Rac1-GTP antibodies. The signal intensity was visualized by a ‘Fire’ color scale using ImageJ. (Positive and negative controls for the detection of active RhoA or Rac1 using this antibody staining are shown in Fig. S5.) Consistent with our rescue experiments, the RhoA activity was decreased in Ptk7 morphants compared to controls (Fig. 6A): calculation of the corrected total cell fluorescence (CTCF) intensity showed a significant decrease in RhoA activity in the whole cells (Fig. 6B) and at cell-cell contacts (Fig. 6C) of Ptk7 morphant NC cells compared to controls. Interestingly, we also observed a change in Rac1 activity. As expected, Rac1 was active in protrusions of control cells, but in Ptk7 morphant NC cells, Rac1-GTP was also detected at cell-cell contacts (Fig. 6A). The fluorescence intensity of the whole cell revealed a significant increase in Rac1 activity in Ptk7 morphant NC cells (Fig. 6B), which was even more evident at cell-cell contacts (Fig. 6C). These results indicate that loss of Ptk7 leads to a decrease in RhoA activity but an increase in Rac1 activity in NC cells.

### Dominant-negative Rac1 restores NC migration in Ptk7 morphants

**Fig. 7.**
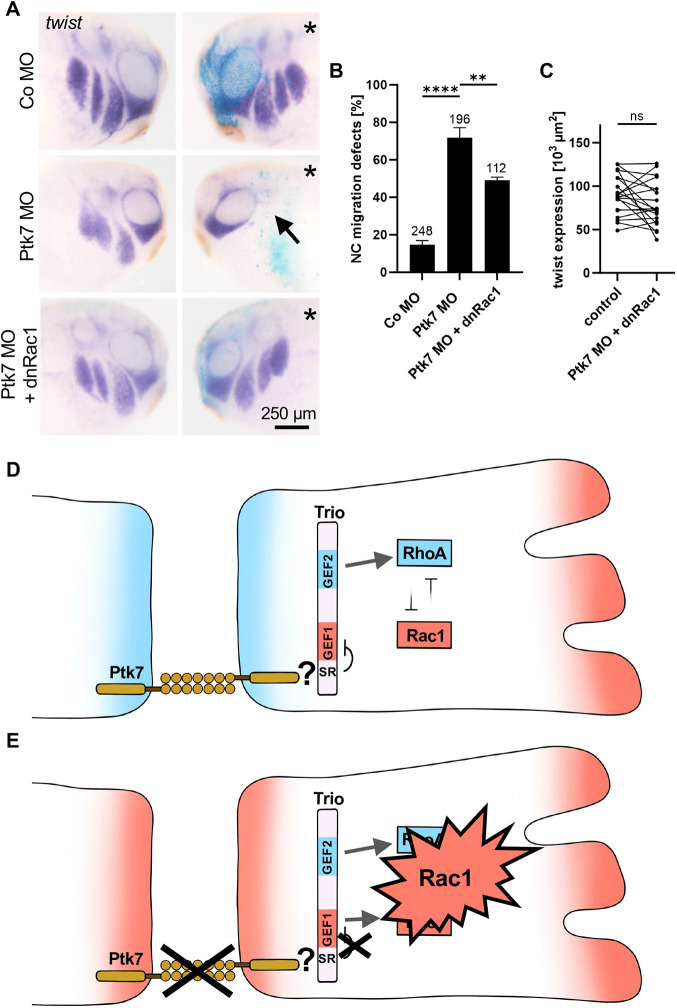
**dnRac1 restores NC migration in Ptk7 morphants.** (A-C) Embryos were injected with 5 ng control MO or 5 ng Ptk7 MO in combination with 50 pg *lacZ* RNA as lineage tracer in one blastomere at the eight-cell stage. Co-injection of 10 pg dnRac1 DNA restored NC migration in Ptk7 morphants. (A) NC cell migration was analyzed at stage 26 by *twist in situ* hybridization. The injected side is marked with an asterisk. NC cell migration defects are indicated by an arrow. Scale bar: 250 µm. (B) Graph summarizing the percentage of embryos with NC migration defects from at least three independent experiments. The number of embryos is indicated for each column. Data are mean±s.e.m. ****P<0.0001, **P<0.01 (one-way ANOVA). (C) Graph showing the measured area of *twist* expression in the branchial arches of 20 randomly selected embryos of the rescue condition. The *twist* expression area of the uninjected side (control) is compared to the injected side of the embryo. ns, not significant (paired *t*-test). (D) At cell-cell contacts, Ptk7 interacts with Trio, thereby inhibiting its GEF1 activity. This is likely achieved by Ptk7 contributing to the autoinhibition of the GEF1 domain, which is mediated by the spectrin repeat domain (SR) of Trio. Thus, the activity of Trio is limited to activation of RhoA, thereby supporting contact inhibition of locomotion. (E) Conversely, loss of Ptk7 function releases this block on the GEF1 domain and leads to hyperactivation of Rac1. Models in D and E were generated using Procreate software.

Ptk7 loss of function leads to an increase in the activity of Rac1 in explanted NC cells. Thus, we next asked whether a dominant-negative construct of Rac1 could rescue the Ptk7 morphant phenotype. Therefore, embryos were injected with Co MO or Ptk7 MO together with *lacZ* RNA as lineage tracer in one dorsal blastomere at the eight-cell stage. For rescue experiments, a dominant-negative form of Rac1 (dnRac1) was co-injected. NC migration was analyzed at tailbud stages by *twist in situ* hybridization. As expected, Ptk7 loss of function severely impaired NC migration compared to the control (Fig. 7A,B). However, co-injection of the dominant-negative Rac1 did indeed restore NC migration in Ptk7 morphants, which was also apparent when the area of *twist* expression was compared between the injected side and non-injected side (Fig. 7C). These data suggest that a Ptk7/Trio complex at NC cell-cell contacts functions by activating RhoA, while inhibiting Rac1.

## DISCUSSION

The RhoGEF Trio is ideally suited to relay signals during cell migration as it contains two distinct GEF domains that activate different members of the Rho GTPase family. In *Xenopus* cranial NC cells, Trio has distinct functions and is required for protrusion formation and migration (Kratzer et al., 2020), but also – as we show here – for CIL. Recently, we found that Trio is transported by microtubules in migrating NC cells (Gossen et al., 2024), suggesting that Trio is dynamically localized to its sites of action, where it may exert different functions. The question is, what are the mechanisms that select the precise Trio activity required at a specific subcellular position?

Here, we take a closer look at the function of Trio at NC cell-cell contacts and identify Ptk7 as a factor that may fine-tune the activity of Trio to functions relevant for CIL. We show that Ptk7 interacts with Trio, and fluorescence complementation assays indicate that this interaction takes place at NC cell-cell contacts. We currently do not know if this interaction is direct, or whether it involves multiple protein domains or even additional interaction partners. Since we recently demonstrated that Ptk7 dynamically accumulates at NC cell-cell contact sites, where it controls CIL (Grund et al., 2021), we analyzed whether Trio is also relevant for this process. Indeed, the repulsive response of Trio morphant NC cells after colliding with another NC cell was reduced compared to controls, suggesting that Trio is required for signaling and cytoskeletal rearrangement during CIL. In support of this, loss of Trio function stabilized microtubules at cell-cell contact zones (Gossen et al., 2024), a phenomenon that has been shown to disrupt CIL behavior (Moore et al., 2013). Interestingly, although Trio controls CIL in single migrating cells, it is not required for protection against NC cell invasion, a process for which Ptk7 is necessary (Grund et al., 2021). This suggests that the upstream signals relevant for NC cell-cell recognition are still intact in Trio morphant cells. Since ectopic expression of the extracellular domain of Ptk7 is sufficient to protect non-NC cell tissue from NC cell invasion (Grund et al., 2021), the presence of cell-cell adhesion molecules, such as Cadherin-11, N-Cadherin or Ptk7, which all mediate CIL (Becker et al., 2013; Grund et al., 2021; Theveneau et al., 2010), might be sufficient to protect Trio morphant NC cells. In contrast, in single colliding NC cells, Trio function is likely required for the response to cell-cell contact formation and the resulting cytoskeletal rearrangement and cell repolarization. Thus, a Ptk7/Trio complex may function in CIL in NC cells by Ptk7 contributing to cell-cell recognition and Trio contributing to the subsequent intracellular signaling response.

How does Ptk7 contribute to the fine-tuning of the activity of Trio at NC cell-cell contacts? Trio is well suited to relay signals to the cytoskeleton as it contains a GEF1 and GEF2 domain that specifically activate Rac1 and RhoA, respectively. Both of these functions seem to be relevant for NC development, because Trio loss of function can be rescued by constitutive active RhoA and Rac1 (Kratzer et al., 2020). In contrast, downstream of Ptk7, Trio appears to function exclusively through its GEF2 domain based on the following: (1) NC migration and protrusion formation of Ptk7 morphants was only restored by the GEF2 domain, but not the GEF1 domain of Trio; (2) Ptk7 loss of function was only rescued by constitutively active RhoA, but not by constitutively active Rac1; (3) Ptk7 loss of function leads to a decrease in RhoA-GTP and to an increase in Rac1-GTP, in particular at NC cell-cell contacts; and (4) dominant negative Rac1 rescues the Ptk7 morphant NC migration phenotype. These data suggest a model in which, at NC cell-cell contacts, Ptk7 may help to limit the GEF activity of Trio to the activation of RhoA by blocking its ability to activate Rac1 (Fig. 7D). Conversely, loss of Ptk7 function causes hyperactivation of Rac1 (Fig. 7E). The molecular mechanism by which this is achieved is unclear, but may involve the spectrin repeat domain. This domain blocks the activity of the GEF1 domain by intramolecular folding, sterically preventing its binding to Rac1 (Bircher et al., 2022; Bonnet et al., 2023). Release of this autoinhibition, either by pathogenic TRIO mutations in humans or by binding of specific cellular interaction partners, leads to Rac1 hyperactivation (Bircher et al., 2022; Bonnet et al., 2023). Thus, during CIL, Ptk7 may contribute to Rac1 inhibition and RhoA activation by interacting with Trio and affecting its catalytic activity. In support of this hypothesis, we noted that co-expression of Ptk7 significantly inhibited the ability of Trio to activate Rac1 (Fig. S6). In contrast to Trio-binding partners such as VE-Cadherin or ICAM-1, which release the intramolecular folding to trigger Rac1 activation (Timmerman et al., 2015; van Rijssel et al., 2012), binding of Ptk7 may enhance the autoinhibition of Trio, possibly by blocking its activators. In addition, Ptk7 may also facilitate the activation of RhoA through the GEF2 domain, as has been show for Gαq, which releases the intramolecular inhibitory contact between GEF2 PH2 and GEF2 DH2, resulting in the activation of RhoA (Lutz et al., 2007; Williams et al., 2007). Thus, at cell-cell contact sites, a Ptk7/Trio protein complex may facilitate GEF2-mediated RhoA activation. This complex may also involve components of the PCP signaling pathway that control CIL by recruiting Dvl to the plasma membrane and locally activating RhoA (Carmona-Fontaine et al., 2008). Indeed, we have previously identified Ptk7 as a regulator of PCP that interacts with Fz and recruits Dvl during NC migration (Lu et al., 2004; Shnitsar and Borchers, 2008). Consistently, we have recently shown that Trio interacts with Dvl (Kratzer et al., 2020), supporting that Dvl may mediate the Ptk7/Trio interaction. Currently, we do not know the molecular mechanisms by which Trio activity is controlled in a Ptk7/Trio complex and whether this involves Dvl, but our data suggest that a Ptk7/Trio complex is relevant for CIL of NC cells.

## MATERIALS AND METHODS

### Constructs

Ptk7 morpholino oligonucleotides (Wehner et al., 2011) and Trio morpholino oligonucleotides (Trio MO2; Kratzer et al., 2020) were used as published. A standard control MO (Gene Tools) was used as a control. In this study, we exclusively used *Xenopus laevis* Ptk7 constructs. The following plasmids were used for RNA or DNA *Xenopus laevis* injection or HEK293 cell transfection: mbGFP (Moriyoshi et al., 1996), mbRFP (Megason and Fraser, 2003), H2B-mCherry (Kashef et al., 2009), Lifeact-RFP (Riedl et al., 2008), H2B-CFP (Hagemann et al., 2014), lacZ (Smith and Harland, 1991), hTRIO-HA (Debant et al., 1996), Ptk7-Myc and ΔkPtk7-Myc (Shnitsar and Borchers, 2008), cPtk7-Myc (Grund et al., 2021) and constitutively active Cdc42 (Schambony and Wedlich, 2007). Constitutively active RhoA, constitutively active Rac1 and dominant negative Rac1 (Schambony and Wedlich, 2007) were recloned into the pCS2+ vector using the indicated restriction sites: constitutively active RhoA (BamH1/EcoR1), constitutively active Rac1 (BamH1/BamH1) and dominant negative Rac1 (BamH1/EcoR1).

For cloning of the *Xenopus laevis* GEF1-HA and GEF2-HA constructs, the coding sequence containing the DH1/2, PH1/2 and SH3 domains were amplified by PCR from cDNA of stage 26 *Xenopus laevis* embryos. N-terminal HA-tagged GEF constructs were generated using the following primers with Cla1/Xho1 restriction sites: XGEF1-forward, 5′CCTAATCGATATGTACCCATACGATGTTCCAGATTACGCTGGTTCCGAAGTGAAGCTTCG3′; XGEF1-reverse, 5′CCTACTCGAGTTAGACAGAGAGGGAATCTTTGTGGT3′; XGEF2-forward, 5′CCTAATCGATATGTACCCATACGATGTTCCAGATTACGCTGGTGACAGTAGTAGCCCATCG3′; XGEF2-reverse, 5′CCATCTCGAGTTAAACTCTGGGGGAGCATCATA3′. The PCR product was cut with ClaI and XhoI, and inserted into the respective sites of pCS2+.

The pCS2+ expression vectors containing split N- and C-YFP fragments were generated by PCR amplification of the N-terminal amino acids 1-157 and the C-terminal amino acids 158-240 of YFP, as previously described (Dottermusch-Heidel et al., 2012), with the exception that a myc- or HA-tag was included in the primer sequence (Table S1). The cloning information for the fluorescence complementation assay constructs is shown in Table S2.

### *Xenopus* microinjection

*Xenopus laevis* embryos were obtained by *in vitro* fertilization and developmental stages were defined according to Nieuwkoop and Faber (Nieuwkoop et al., 1956). All procedures were performed in accordance with the German animal use and care law (Tierschutzgesetz) and approved by the German state administration Hesse (Regierungspräsidium Giessen, V 7/2022). Sense-capped mRNA was synthesized *in vitro* using the SP6 mMESSAGE mMACHINE System (Thermo Fisher Scientific). Embryos were injected into one blastomere at the two- or eight-cell stage, as indicated.

### Whole-mount *in situ* hybridization

Embryos were cultured until neurula (stage 17/18) and tadpole stages (stage 26-28), fixed with MEMFA (3.7% formaldehyde, 0.1 M MOPS, 2 mM EGTA and 2 mM MgSO_4_) and further analyzed using β-galactosidase staining and *in situ* hybridization (Harland, 1991; Smith and Harland, 1991) using the NC marker *twist*. The investigators were blinded to the group allocation when evaluating the experimental outcome. Phenotypic images were taken with a Nikon stereo microscope (SMZ18) with a DS-Fi3 Nikon camera and NIS-Elements imaging software. The area of the *twist* expression was measured manually by using the ImageJ polygon selection tool. GraphPad Prism 9 was used for statistical analysis.

### NC cell explants and analysis

NC explants were dissected at stage 17-19, transferred to a fibronectin-coated (Sigma-Aldrich, 1:100) chamber slide or coverslip and cultured in 0.8×MBS [10 mM HEPES (pH 7.0), 88 mM NaCl, 1 mM KCl, 2.4 mM NaHCO_3_, 0.82 mM MgSO_4_, 0.41 mM CaCl_2_ and 0.66 mM KNO_3_) for at least 1 h to ensure adherence of the cells. Images of NC explants were taken with a Zeiss Spinning Disk system (Axio Observer Z1) and analyzed using Zen blue or ImageJ software. Cell circularity was determined by ImageJ using the formula 4 π (area/perimeter^2^). Statistical analysis was performed using GraphPad Prism 9.

For the single cell collision assay, NC cells were cultured for 4 h at room temperature. Time-lapse imaging was started at the time point when the NC cells migrated as single cells. The coordinates of the NC cells before (−Δt), during (t=0) and after (+Δt) cell-cell contact were determined by manual tracking using ImageJ. Only single migrating cells that showed no interaction ∼10 min before and after the cell-cell collision were used for the analysis. The initial velocity vector (red, Fig. 4B) was defined and its orientation standardized as follows: the starting point describes the initial position of the cell at the beginning of the analysis (−Δt) and the end point marks the position of the cell at the time of collision (t=0). Black arrows in Fig. 4B indicate the direction of migration after collision. Changes in the migration direction of individual NC cells after cell collision were analyzed using a custom MATLAB script, as previously described (Carmona-Fontaine et al., 2008), with the addition that the distribution of relative velocity vectors were shown in a rose plot and that the angle between the initial vector and the velocity vector were displayed. For quantification, all velocity vectors that were below the initial vector and thus had an angle between 180° and 360°, were mirrored horizontally so that all angles were between 0° and 180°.

For confrontation assay, control and MO-injected NC explants were placed in close proximity. The overlap area (white, Fig. 4D) at the time point of maximum overlap (Δt) was measured and the overlap index (OI) was calculated using a custom MATLAB script (Becker et al., 2013).

For immunostaining, NC cells were fixed with glyoxal (19.7% ethanol, 3.1% glyoxal and 0.75% acetic acid in H_2_O) for 20 min. Subsequently, the explants were washed three times for 10 min each with 1×PTw (1×PBS and 0.1% Tween) and incubated in blocking solution [10% FCS (Sigma-Aldrich), 1% penicillin/streptomycin (Sigma-Aldrich) in PTw] for 1 h at room temperature. The primary antibody [anti-active Rac1-GTP (Cao et al., 2015; Shellard and Mayor, 2021), NewEast Biosciences, NB-26903; or anti-active RhoA-GTP (Cao et al., 2015), NewEast Biosciences, NB-26904] was diluted 1:100 in blocking solution and incubated overnight at 4°C. After washing three times for 10 min each with 1×PTw, the secondary antibody (anti-mouse 488, Life Technologies, A-11029) was diluted 1:400 in blocking solution and incubated for 3 h at room temperature. The explants were washed three times for 10 min each with 1×PTw and the cover slips were mounted on microscope slides using mounting medium (Dako). The fluorescence intensity was measured by calculating the corrected total cell fluorescence (CTCF) (Gavet and Pines, 2010). The selection used for measurement was determined using ImageJ by manually selecting the cell boundary (whole cell) or by placing a 10 µm wide rectangle over the cell-cell contact. The average background intensity was calculated from three random recordings of the background.

### Cell transfection and co-immunoprecipitation

HEK293 cell cultivation and transfection were performed as previously described (Kratzer et al., 2020) using jetPEI (Polyplus, Graffenstaden, France) as transfection reagent. HEK293 cells were tested regularly for mycoplasma contamination. Co-immunoprecipitations (co-IP) with Dynabeads Protein G beads (Thermo Scientific) were performed according to the manufacturer's protocol using anti-myc antibodies (Abcam, ab9132, 1:250). Antibody-resin coupling was carried out for 10 min and the antigen-antibody reaction for 20 min. For the Trio co-IP, an indirect method was used: protein lysates were incubated with the antibody anti-HA (Abcam, ab9110, 1:100) on an end-over-end rotator for 2 h at 4°C. Afterwards, 40 µl Dynabeads were added and incubated for 30 min at 4°C. The beads were washed three times with 500 μl 0.02% Tween-20/PBS and eluted in 20 μl 6× Laemmli sample buffer [350 mM Tris-HCl (pH 6.8), 9.3% dithiothreitol, 30% glycerin, 10% SDS and 0.02% Bromophenol Blue].

For co-IPs with Protein G-Agarose (Roche), 500 µl IP buffer [50 mM Tris (pH 7.5), 150 mM NaCl, 0.5% Triton X-100 and 0.5% NP-40] containing phosphatase and protease inhibitor cocktail tablets (Roche), 25 µl agarose beads and anti-Myc antibodies (Abcam, ab9132, 1:300) were incubated on an end-over-end rotator for 30 min at 4°C. Agarose-antibody complexes were collected by centrifugation at 3000 ***g*** for 1 min in a microcentrifuge at 4°C. Afterwards, the beads were washed with 500 µl IP buffer, centrifuged again and the supernatant was discarded. The protein lysates were added and incubated for 2 h at 4°C on an end-over-end rotator. Agarose-antibody-antigen complexes were collected by centrifugation for 1 min at 3000 ***g*** in a microcentrifuge at 4°C. Subsequently, the complexes were washed four times using 500 µl IP buffer. Each washing step was followed by centrifugation and removal of the supernatant. Samples were eluted in 25 µl 6× Laemmli sample buffer.

For western blot analysis, the following antibodies were used: anti-Myc (Abcam, ab9132, 1:1000), anti-HA (Abcam, ab9110, 1:1000), anti-Myc (Cell Signaling Technology, 2276, 1:1000), anti-rabbit-800 (LI-COR, 926-32213, 1:7500), anti-mouse-680 (LI-COR, 926-32212, 1:7500), anti-goat-680 (LI-COR, 926-68074, 1:7500), anti-goat-HRP (Santa Cruz, sc-2020, 1:5000) and anti-rabbit-HRP (Cell Signaling Technology, 7074S, 1:2000).

## Supplementary Material



10.1242/develop.204446_sup1 Supplementary information
