# Supplementary material for: The Rho GEF Trio functions in contact inhibition of locomotion of neural crest cells by interacting with Ptk7
Source: Development. 2025 May 6;152(9):dev204446. doi: 10.1242/dev.204446 (PMC12083869; doi:10.1242/dev.204446)
Supplement: Supplementary information [file develop-152-204446-s1.pdf]

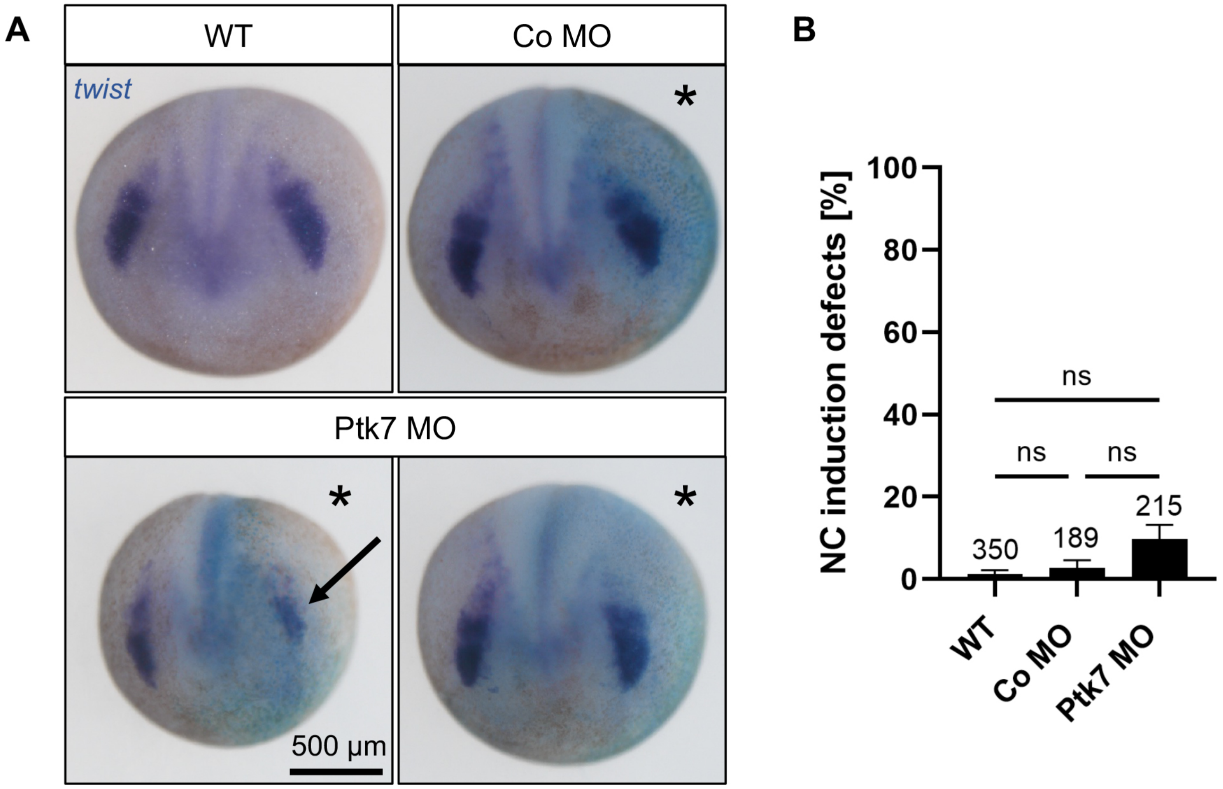


**Fig. S1. Ptk7 loss-of-function does not affect NC induction.** Two-cell stage embryos were injected with 7.5 ng Ptk7 MO or a control MO in combination with 75 pg *lacZ* RNA as lineage tracer. **(A)** NC cell induction was analyzed at stage 17/18 by *twist in situ* hybridization. Asterisks mark the injected side. Arrow indicates NC induction defect. Scale bar: 500 µm. **(B)** Graph showing NC induction defects of four independent experiments. The total number of embryos is indicated for each column. Data are mean ±s.e.m., ns = not significant (one-way ANOVA).

Development • Supplementary information

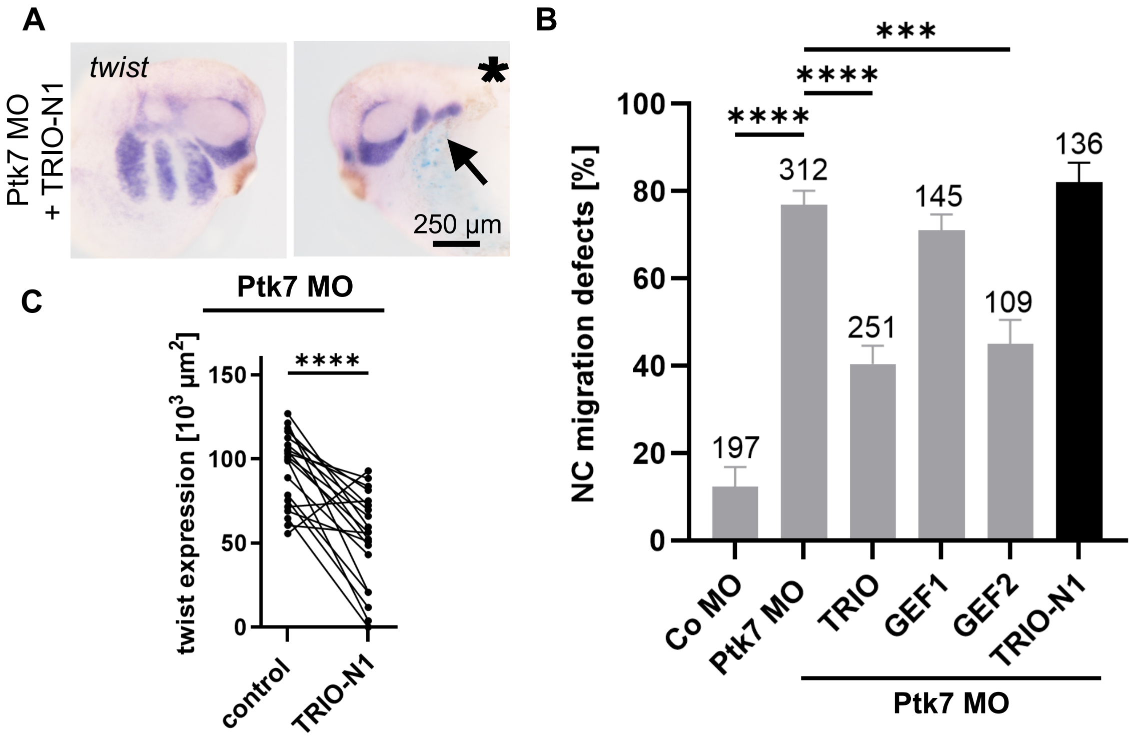


**Fig. S2. TRIO-N1 does not rescue NC migration in Ptk7 morphants.** Two-cell stage embryos were injected with 6.5-7.5 ng Ptk7 MO or a control MO in combination with 75 pg *lacZ* RNA as lineage tracer. Co-injection of 150 pg TRIO DNA and 100 pg *GEF2* RNA, but not 100 pg *GEF1* RNA or 100 pg TRIO-N1 DNA, restored NC cell migration in Ptk7 morphants. The data shown here include the data already shown in Fig. 1. **(A)** NC cell migration was analyzed at stage 26 by *twist in situ* hybridization. Asterisks mark the injected side. Arrow indicates NC migration defects. Scale bar: 250 µm. **(B)** Graph summarizing the percentage of embryos with NC migration defects of at least three independent experiments. The total number of embryos is indicated for each column. Data are mean ±s.e.m. **** $p<0.0001$, *** $p<0.001$ (one-way ANOVA). **(C)** Quantification of the *twist in situ* hybridization shown in (A). Graph presenting the measured area of *twist* expression of 20 randomly selected embryos of the rescue condition using TRIO-N1. The *twist* expression of the uninjected side (control) is compared to that of the injected side of the embryo. **** $p<0.0001$ (paired t-test).

Development • Supplementary information

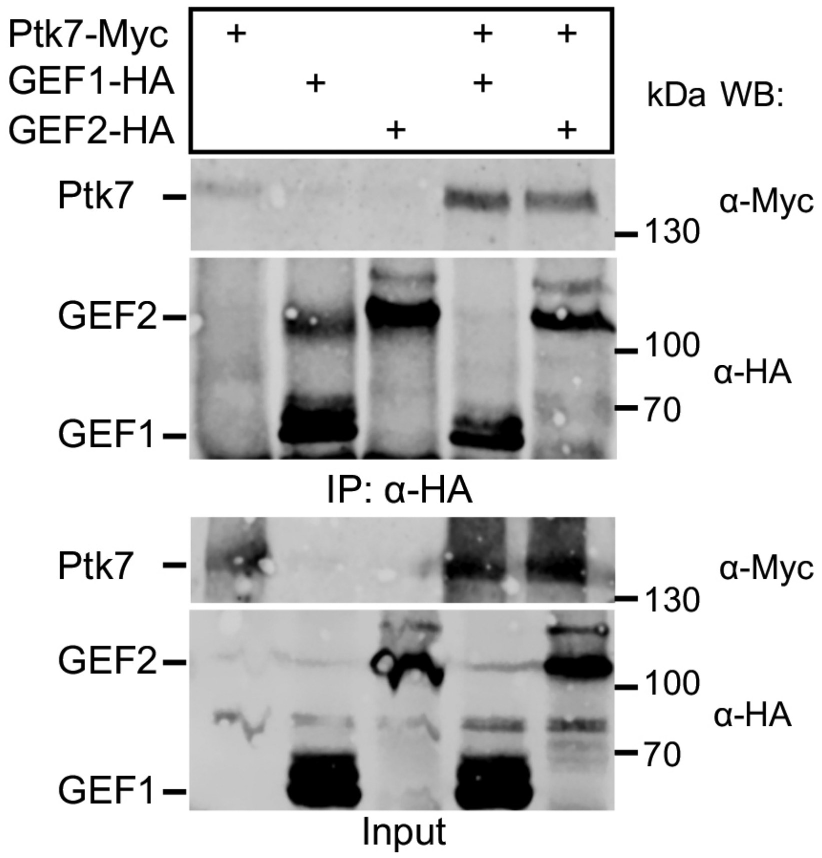


**Fig. S3. The Trio GEF domains interact with Ptk7 in HEK293 cells.** HEK293 cells were transfected as indicated and immunoprecipitations were performed using an anti-HA antibody. Co-immunoprecipitated proteins are shown in the upper panel, immunoprecipitated proteins in the middle panel and cell lysates in the bottom panel. Antibodies used for Western blotting and molecular weights (kDa) are indicated on the right. Representative results of at least three independent experiments are shown.

Development • Supplementary information

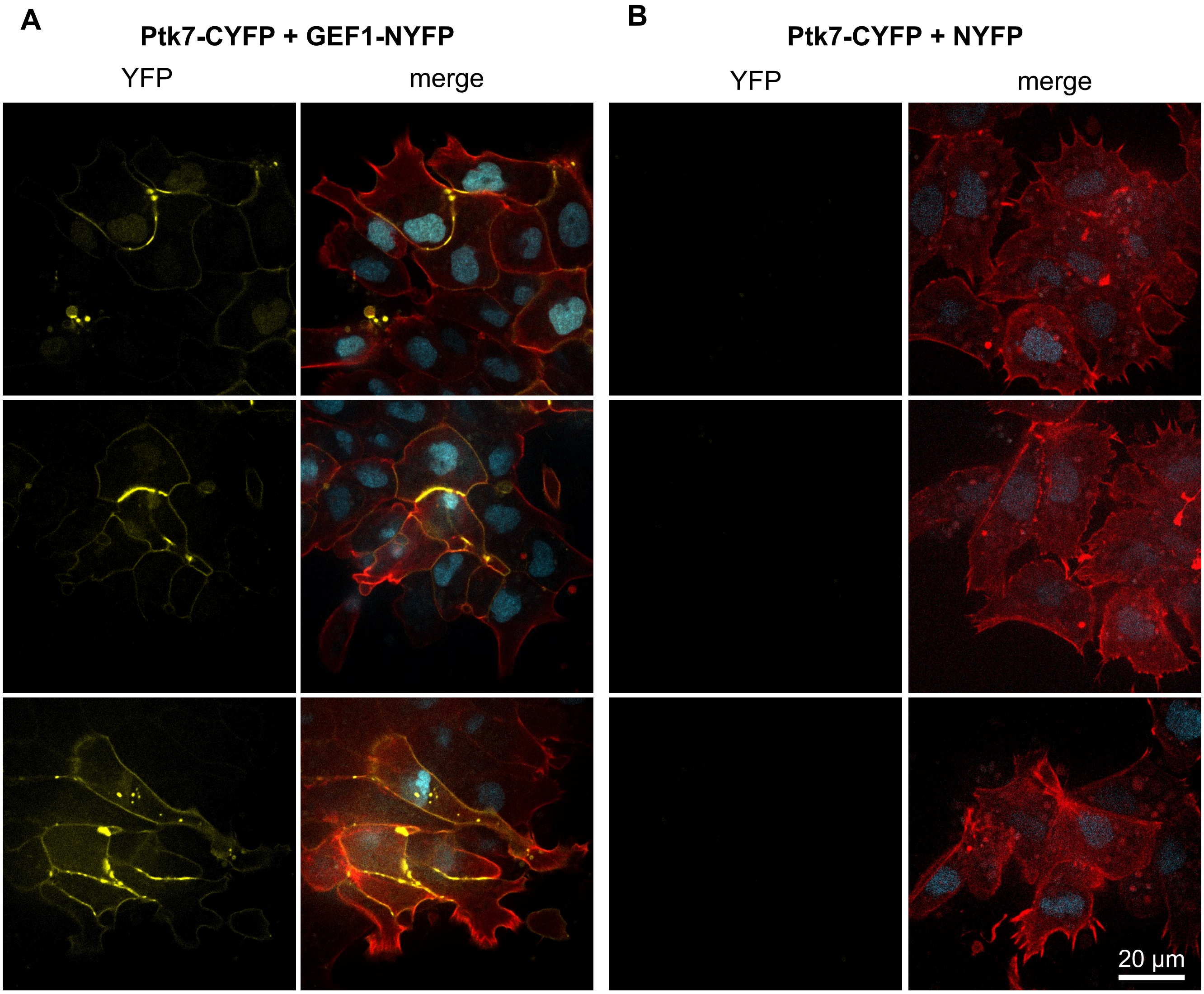


**Fig. S4. PTK7-CYFP complements with GEF1-NYFP, but not NYFP alone.** NC explants were injected with 100 pg *Ptk7-CYFP* RNA in combination with 100 pg *GEF1-NYFP* RNA or 100 pg *NYFP* RNA together with 200 pg *H2B-CFP* RNA and 300 pg *lifeact-RFP* RNA in one blastomere at the two-cell stage. For each combination three representative images are shown. YFP signal indicates an interaction of Ptk7 and GEF1 at NC cell-cell contacts, while no signal is detected in explants expressing Ptk7-CYFP and NYFP using identical imaging parameters. Scale bar: 20 µm.

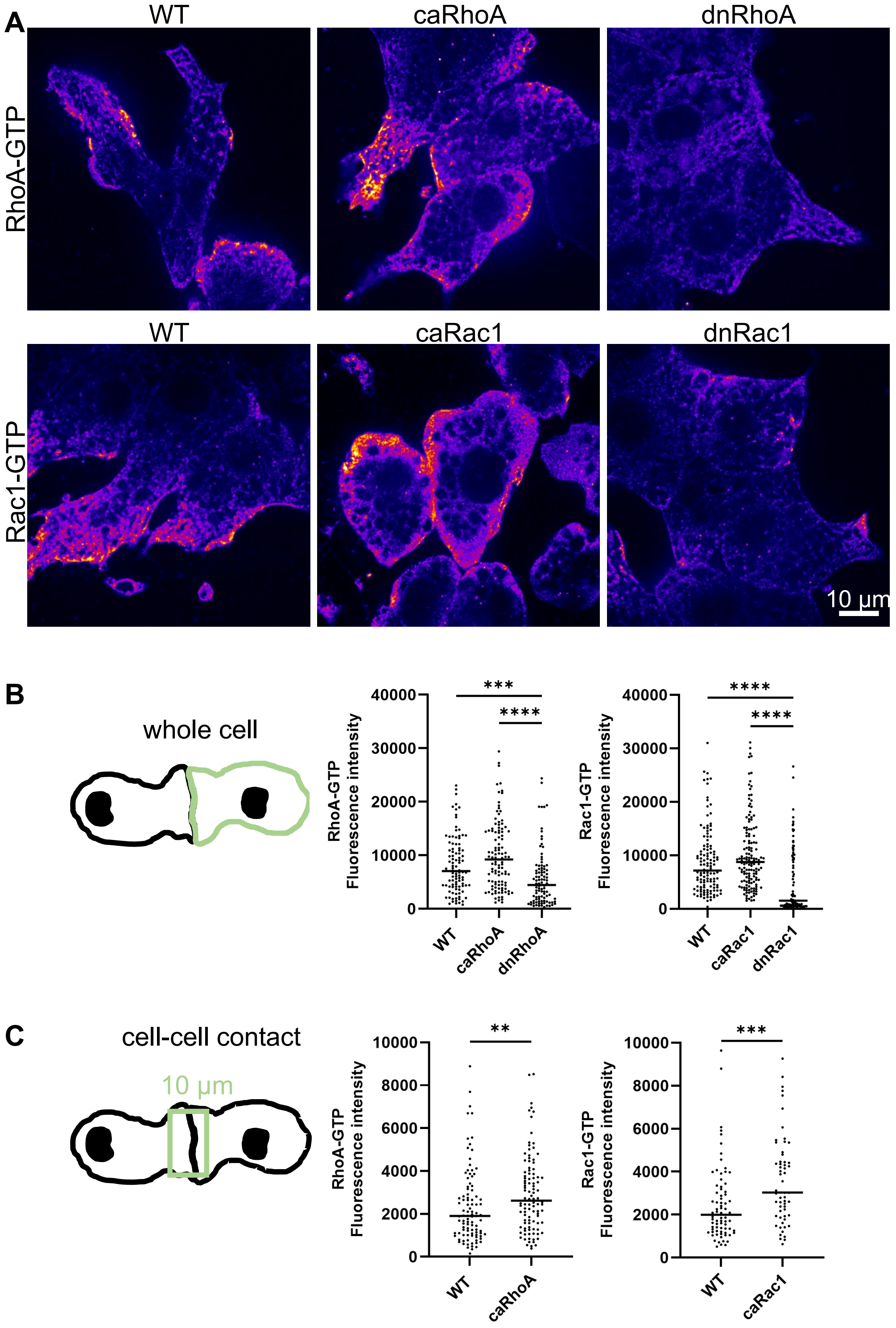


Development • Supplementary information

**Fig. S5. Positive and negative controls for the detection of active RhoA and Rac1 by antibody staining. (A)** Embryos were injected with 10-20 pg caRhoA, dnRhoA, caRac1, dnRac1 DNA in combination with 200 pg *mbRFP* RNA as lineage tracer in one blastomere at the 8-cell stage. NC cells were explanted at stage 18 and fixed after three hours of cultivation. An antibody staining against RhoA-GTP or Rac1-GTP was performed. The ’Fire’ lookup table of ImageJ was applied. Scale bar: 10 µm. **(B)** Quantification of the antibody signal of the whole cell. The CTCF is plotted, the median is indicated as a line. ****$p<0.0001$, ***$p<0.001$ (Kruskal-Wallis test). **(C)** Fluorescence intensity at NC cell-cell contact sides. The CTCF was determined in a square with a width of 10 µm that was placed over the entire length of the cell-cell contact (see cartoon). The median CTCF of at least three independent experiments is blotted as a line. **$p<0.01$, ***$p<0.001$ (Mann-Whitney test).

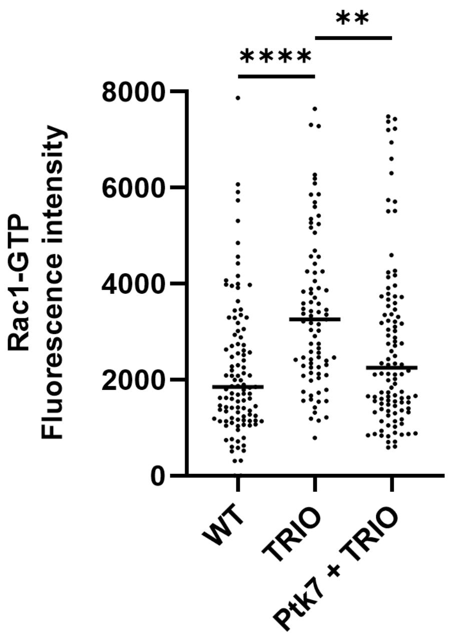


**Fig. S6. Co-expression of Ptk7 inhibits Trio's ability to activate Rac1.** Embryos were injected with 150 pg TRIO-HA DNA and 250 pg *mRFP* RNA alone or in combination with 250 pg *Ptk7-Myc* RNA. NC cells were explanted at stage 18 and fixed after three hours of cultivation. An antibody staining against Rac1-GTP was performed and the CTCF was determined at the cell-cell contact side. The median CTCF of two independent experiments is indicated as a line. **p<0.01, ****p<0.0001 (Kruskal-Wallis test).

## Table S1.

| Primer | Sequence (5'→3') |
|---|---|
| NYFP-HA-forward | TACTGGATCCATGGTGAGCAAGGGCGAGGA |
| NYFP-HA-reverse | TAATCTCGAGTGCGTAATCCGGTACATCGTAAGGGTACTTGTCGGCGGTGATATAGAC |
| HA-NYFP-forward | TACTCTCGAGTACCCTTACGATGTACCGGATTACGCAGTGAGCAAGGGCGAGGAGCT |
| HA-NYFP-reverse | TAATTCTAGATTACTTGTCGGCGGTGATAT |
| MT-CYFP-forward | AGCTCTCGAGGAACAAAAACTCATCTCAGAAGAGGATCTGCAGAAGAACGGCATCAAGGT |
| MT-CYFP-reverse | GAATTCTAGATTACTTGTACAGCTCGTCCATGC |

## Table S2.

| Split-YFP plasmid | Insert vector | PCR amplified insert | Acceptor vector | Primer sequence (5'→3') |
|---|---|---|---|---|
| Ptk7-Myc-CYFP/ pCS2+ | *Xenopus laevis* Ptk7-Myc/ pCS2+ (Shnitsar and Borchers, 2008) | *Xenopus laevis* Ptk7 | Myc-CYFP/ pCS2+ | Forward: TTGGATCCATGGGGCCGATTGTGCTC<br>Reverse: CCATCTCGAGCCCTTGTGTCTTGCTGCC |
| GEF1-HA-NYFP/ pCS2+ | *Xenopus laevis* GEF1-HA /pCS2+ | *Xenopus laevis* GEF1 | HA-NYFP/ pCS2+ | Forward: CTAGATCGATATGGGTTCCGAAGTGAAG<br>Reverse: TATACTCGAGGACAGAGAGGGAATCTTT |
| GEF2-HA-NYFP/ pCS2+ | *Xenopus laevis* GEF2-HA /pCS2+ | *Xenopus laevis* GEF2 | HA-NYFP/ pCS2+ | Forward: GAGTATCGATATGGGTGACAGTAGTAGCC<br>Reverse: CTAGCTCGAGCAACTCTGGGG |
| NYFP-HA-TRIO/ pEGFP-C1* | NYFP-HA/ pCS2+ | NYFP-HA | TRIO/ pEGFP-C1 | Forward: TACTACCGGTATGGTGAGCAAGGGCGA<br>Reverse: AGCTTCCGGATGCGTAATCCGGTACATC |

*GFP was cut out and removed by digestion with AgeE1 and BspE1.

Development • Supplementary information
