# Supplementary material for: The Rho GEF Trio functions in contact inhibition of locomotion of neural crest cells by interacting with Ptk7
Source: Development. 2025 May 6;152(9):dev204446. doi: 10.1242/dev.204446 (PMC12083869; doi:10.1242/dev.204446)
Supplement: Supplementary file 1 [file develop-152-204446_review_history.pdf]

The Rho GEF Trio functions in contact inhibition of locomotion of neural crest cells by interacting with Ptk7

Katharina Till and Annette Borchers
DOI: 10.1242/dev.204446

Editor: Thomas Lecuit

Review timeline
Original submission: 11 October 2024
Editorial decision: 18 December 2024
First revision received: 11 March 2025
Accepted: 7 April 2025

Original submission

First decision letter

MS ID#: dev.204446

MS Title: The Rho GEF Trio functions in contact inhibition of locomotion of neural crest cells by interacting with Ptk7

Authors: Annette Borchers; Katharina Till
Article Type: Research Article

Dear Dr Borchers,

I have now received all the referees reports on the above manuscript, and have reached a decision. The referees' comments are appended below, or you can access them online: please go to .

The overall evaluation is positive and we would like to publish a revised manuscript in Development, provided that the referees' comments can be satisfactorily addressed. Please attend to all of the reviewers' comments in your revised manuscript and detail them in your point-by-point response. If you do not agree with any of their criticisms or suggestions explain clearly why this is so. If it would be helpful, you are welcome to contact us to discuss your revision in greater detail. Please send us a point-by-point response indicating your plans for addressing the referees' comments, and we will look over this and provide further guidance.

Reviewer 1

Advance summary and potential significance to field

This study examines a role of the interaction of the pseudokinase PTK7 and Trio, a molecule that activates Rac and Rho through two distinct GEF domains, in neural crest migration in Xenopus embryos. The authors' prior work implicated both proteins in neural crest development, and they are reporting that the two molecules functionally and physically interact. Pull downs from transfected HEK293T cell lysates and YFP complementation assays in embryonic cells demonstrate the interaction of PTK7 with Trio constructs along the cell-cell contacts. The authors suggest a novel mechanism in which the association with PTK7 favors the Trio RhoGEF activity at cell junctions, while suppressing Rac1 activation.

© 2025. Published by The Company of Biologists under the terms of the Creative Commons Attribution License (https://creativecommons.org/licenses/by/4.0/).

Comments for the author

Since the proposed mechanism is novel and important, the publication is recommended if the authors are able to provide further support to their hypothesis as detailed below.

Major: In Fig 5, immunostaining is used to assess RhoA versus Rac1 activation, using specific antibodies. This is an important approach that would benefit from positive and negative controls and the normalization to a membrane marker. Importantly, the same analysis can be used to test the main hypothesis that PTK7 binding to Trio restricts its activity to RhoA rather than Rac1. For example, in a gain-of-function study, Trio alone would be expected to activate both Rac-GTP and Rho-GTP, while in the presence of PTK7, Trio may preferentially increase Rho-GTP over Rac-GTP. Alternatively, the selective activation of RhoA vs Rac1 following Trio and PTK7 expression could be shown in the biochemical assay that they previously used (Kratzer et al., 2020; PMID: 32366678) or with the Rho activity sensor mCherry-2xrGBD (Chumki et al., 2022; PMID: 36200892).

Other comments:

-Since all constructs appear to interact in the YFP complementation assay, a negative control using NYFP plus Ptk7-CYFP is warranted.
-Some YFP complementation (Fig. 2D) and Rho activation (Fig. 5) signals are observed outside cell-cell contacts. It would help to see images with larger fields of view (15-20 cells each).
- In Fig. 2A, all tested PTK7 constructs are detected in Trio-HA pulldowns, leading to the proposal that PTK7 juxtamembrane domain is critical for the binding. A direct test of this idea after the deletion of the putative interaction domain would strengthen the paper, or alternative explanations can be offered, e. g. multiple interacting domains.
- The Trio band in the HA pulldown is faint, raising concerns about assay specificity, as a similar band appears in the control (Fig. 2A, lanes 3 and 4), and Trio-HA expression in lysates is not shown. Can this be improved?
--Fig. 3 aims to show that Trio is required for the "contact inhibition of locomotion". On the other hand, the invasion was not affected in morphant cells. Please comment on whether Trio depletion affects the frequency of cell-cell collisions, the time of cell-cell contact and the percent of the cell perimeter involved in the contact. How was the initial velocity vector (red arrow) defined? Given the complexity of these assays, the authors should acknowledge that more than one interpretation of their results is possible.

-- The partial rescue of neural crest migration of PTK7 morphants by caRhoA, caRac1 and caCdc42 (Fig. 4) seems at odds with the rescue shown with dnRac (Fig. 6) and deserves a comment.

--Does the association of PTK7 with Trio affect the binding of Trio to other targets that modulate NCC migration, including Dvl, Cadherin 11 or Par3? While it is not directly relevant, this knowledge may provide further support to the proposed mechanism.

Reviewer 2: This manuscript explores the role of the Rho GEF Trio during Xenopus neural crest migration. Specifically, the authors analyze how its interaction with Ptk7 could regulate contact inhibition of locomotion. They demonstrate that the inhibition of neural crest migration caused by blocking Ptk7 can be rescued by Trio, specifically through the GEF2 domain of the protein. Using co-immunoprecipitation and bimolecular fluorescent complementation assays, they show that Ptk7 interacts with Trio, apparently through the GEF1 and GEF2 domains. Additionally, they confirm that Trio inhibition impairs contact inhibition of locomotion using a single-cell assay, but not when an explant invasion assay is employed. Through a series of epistatic experiments, they provide evidence that Trio acts upstream of RhoA during neural crest migration, which in turn modulates Rac activity.

This is an elegant and well-executed study that identifies the molecular mechanisms by which contact inhibition of locomotion drives the directional migration of neural crest cells in vivo. Contact inhibition of locomotion was discovered 70 years ago, but its molecular basis has remained elusive. This work connects key molecules, including Trio, Ptk7, RhoA, and Rac, as regulators of this process. The experiments are well-designed with a clear rationale, and the data are convincing.

© 2025. Published by The Company of Biologists under the terms of the Creative Commons Attribution License (https://creativecommons.org/licenses/by/4.0/).

I have only minor suggestions that could enhance the impact of this work:

1. Localized vs. Global Rac1 Inhibition:
The model proposed by the authors suggests a localized regulation of RhoA and Rac at the cell contact, consistent with previous studies (e.g., Carmona-Fontaine et al., 2008). However, in Fig. 6, they demonstrate that inhibition of Ptk7 is rescued by global (not localized) inhibition of Rac1. How is this possible? How does the inhibition of Rac1 throughout the cell, including protrusions known to be Rac1-dependent, not impair neural crest migration? A potential experiment to address this question would be to analyze cell protrusions using the neural crest explant assays shown in other figures. Perhaps cell protrusions are not completely inhibited in the dnRac1, and this residual activity is sufficient to drive directional migration in the embryo.

2. Twist Expression and Neural Crest Migration:
Some experiments described as showing inhibition of neural crest migration also report reduced expression of Twist in neural crest cells. How do the authors rule out the possibility that impaired migration is a consequence of a reduced number of neural crest cells (fewer cells expressing Twist) due to an early effect on neural crest formation or induction? To address this, the authors could examine Twist expression, or another neural crest marker, at earlier (premigratory) stages.

3. Bimolecular Fluorescent Complementation Controls:
The bimolecular fluorescent complementation assay effectively demonstrates the interaction between Ptk7 and Trio. However, the controls (with the individual molecules), which are currently presented in the supplementary figure, should be included in the main figure. These controls are critical for correctly interpreting the data.

4. Contact Inhibition of Locomotion vs. Invasion Assay:
The authors report a clear effect of Trio inhibition on contact inhibition of locomotion, but no effect on the invasion assay (Fig. 3D). This is somewhat puzzling, as these assays are typically consistent. Since this is a negative result, including a positive control that demonstrates increased invasion after impairing contact inhibition of locomotion would strengthen the conclusion. However, I do not consider this essential as long as the data (number of experiments, statistical analysis, etc.) for Fig. 3D are robust.

First revision

<u>Author response to reviewers' comments</u>

Response to the Reviewers' comments to the manuscript dev.204446

We thank the reviewers for their constructive criticism that helped us to improve our manuscript. In the following we will briefly state how we have addressed the respective comments:

Response to Reviewer 1

1. Major: In Fig 5, immunostaining is used to assess RhoA versus Rac1 activation, using specific antibodies. This is an important approach that would benefit from positive and negative controls and the normalization to a membrane marker.

We agree with the reviewer and have now included **positive and negative controls** (supplementary **Fig. S5**). We injected constitutive active (ca) and dominant negative (dn) RhoA or Rac1, respectively, performed immunostaining, and analyzed the fluorescence intensity by determining the corrected total cell fluorescence (CTCF). As expected, we observed a significant decrease in RhoA or Rac1 activity, when the dominant negative constructs were injected (Fig. S5A,B). Conversely, constitutive active constructs led to an increase, although the effect was not significant compared to controls when we analyzed whole NC cells (Fig. S5A,B). Possibly RhoA and

© 2025. Published by The Company of Biologists under the terms of the Creative Commons Attribution License (https://creativecommons.org/licenses/by/4.0/).

Rac1 activity in wild-type NC cell were already relatively high so that it was difficult to detect a significant increase. Another problem is that we cannot use higher concentrations of the constitutive active constructs as these cause developmental defects. Therefore, we turned to the cell-cell contact side, where we expect the Ptk7/Trio complex to function. Indeed, here we detected a significant increase in RhoA and Rac1 activity in response to the constitutive active constructs compared to the uninjected control (Fig. S5C).

When we calculated the CTCF, we **normalized** to the average background intensity, which was calculated from three random positions. Normalization to a membrane marker is an option, but relies on a good antibody detecting a membrane protein, which is challenging in *Xenopus* embryos. Furthermore, we find normalization to the background intensity more appropriate, because we either calculate the CTCF from whole cells or a 10 µm wide rectangle covering the cell-cell contact side. Thus, the membrane makes up only a fraction of the area that we measure. Out of curiosity, we tested whether the fluorescence intensity of a membrane targeted protein (mRFP) would potentially be affected by expression of constitutively active or dominant negative Rac1, but we did not find any evidence for this (figure for the reviewer **Fig. R1**).

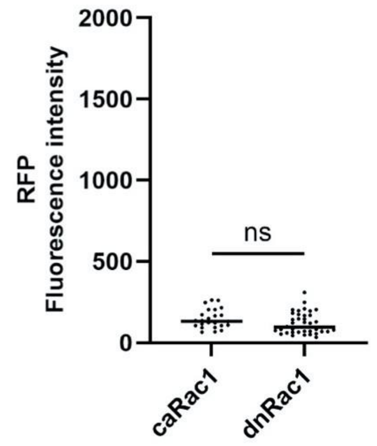

Fig. R1 mRFP fluorescence in NC cells at cell-cell contact sites expressing caRac1 and dnRac1.

© 2025. Published by The Company of Biologists under the terms of the Creative Commons Attribution License (https://creativecommons.org/licenses/by/4.0/).

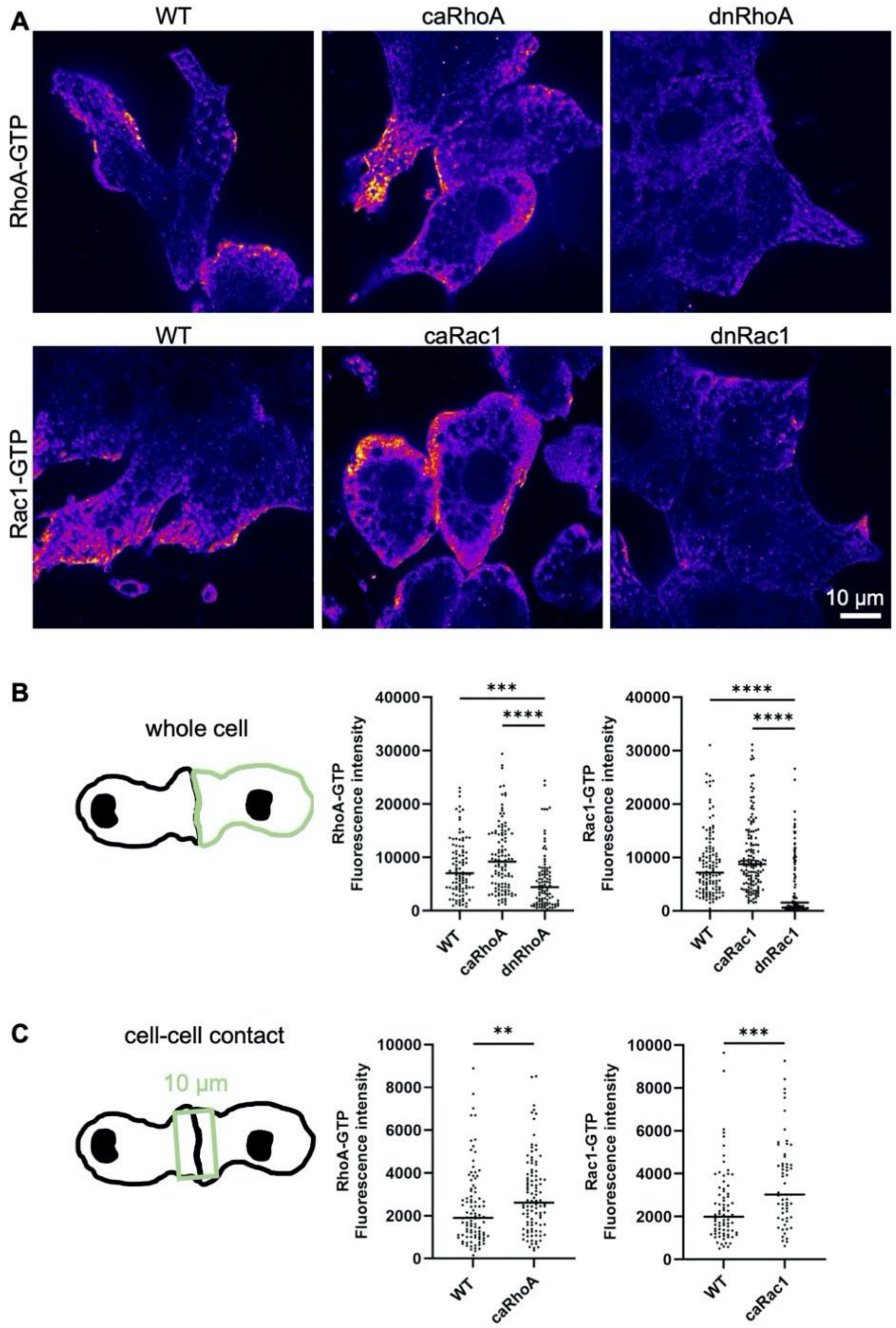

Supplementary Fig. S5: Positive and negative controls for the detection of active RhoA and Rac1 by antibody staining. (A) Embryos were injected with 10-20 pg caRhoA, dnRhoA, caRac1, dnRac1 DNA in combination with 200 pg *mbRFP* RNA as lineage tracer in one blastomere at the 8-cell stage. NC cells were explanted at stage 18 and fixed after three hours of cultivation. An antibody staining against RhoA-GTP or Rac1-GTP was performed. The 'Fire' lookup table of ImageJ was applied. Scale bar: 10 μm. (**B**) Quantification of the antibody signal of the whole cell. The corrected total cell fluorescence (CTCF) is plotted, the median is indicated as a line. ****p<0.0001, ***p<0.001 (Kruskal-Wallis test). **(C)** Fluorescence intensity at NC cell-cell contact sides. The CTCF was determined in a square with a width of 10 μm that was placed over the entire length of the cell-cell contact (see cartoon). The median CTCF of at least three independent experiments is blotted as a line. **p<0.01, ***p<0.001 (Mann-Whitney test).

© 2025. Published by The Company of Biologists under the terms of the Creative Commons Attribution License (https://creativecommons.org/licenses/by/4.0/).

2. Importantly, the same analysis can be used to test the main hypothesis that PTK7 binding to Trio restricts its activity to RhoA rather than Rac1. For example, in a gain-of-function study, Trio alone would be expected to activate both Rac-GTP and Rho-GTP, while in the presence of PTK7, Trio may preferentially increase Rho-GTP over Rac-GTP. Alternatively, the selective activation of RhoA vs Rac1 following Trio and PTK7 expression could be shown in the biochemical assay that they previously used (Kratzer et al., 2020; PMID: 32366678) or with the Rho activity sensor mCherry-2xrGBD (Chumki et al., 2022; PMID: 36200892).

We thank the reviewer for this suggestion. Our central hypothesis is that in the presence of Ptk7, Trio's ability to activate Rac1 is reduced. As Trio already activates RhoA and since we had difficulties to significantly increase RhoA activation with the constitutive active constructs (see reviewer question 1), we did not attempt to detect a potential increase in RhoA activity in the presence of Ptk7. However, we tested the effect of Ptk7 on the Trio-mediated activation of Rac1.

To analyze how Ptk7 modulates Trio's ability to activate Rac1 we focused on the cell-cell contact zones, where we expect this interaction to be relevant. Therefore, we used the immunostaining approach, which allows us to distinguish subcellular areas rather than our previous biochemical approach. Indeed, supporting our hypothesis, TRIO overexpression significantly activated **Rac1 expression**, an effect that was **significantly decreased when Ptk7 was co- expressed** (supplementary **Fig.S6**). This point has now been added to the discussion (line 443-445).

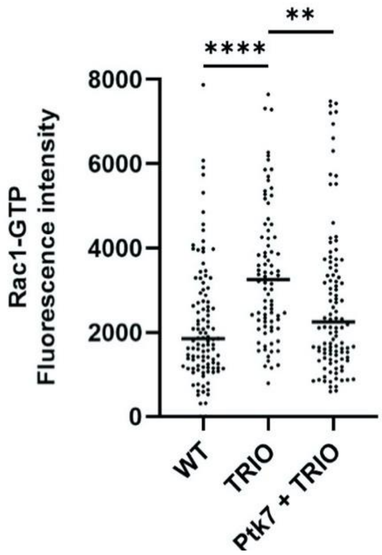

Supplementary Fig. S6: Co- expression of Ptk7 inhibits Trio's ability to activate Rac1. Embryos were injected with 150 pg TRIO-HA DNA and 250 pg *mRFP* RNA alone or in combination with 250 pg *Ptk7-Myc* RNA. NC cells were explanted at stage 18 and fixed after three hours of cultivation. An antibody staining against Rac1-GTP was performed and the CTCF was determined at the cell-cell contact side. The median CTCF of two independent experiments is indicated as a line.
$^{**}p<0.01$, $^{****}p<0.0001$ (Kruskal- Wallis test).

3. Since all constructs appear to interact in the YFP complementation assay, a negative control using NYFP plus Ptk7-CYFP is warranted.

We thank the reviewer for this comment and have now added this control in a new supplementary figure (**Fig. S4**).

© 2025. Published by The Company of Biologists under the terms of the Creative Commons Attribution License (https://creativecommons.org/licenses/by/4.0/).

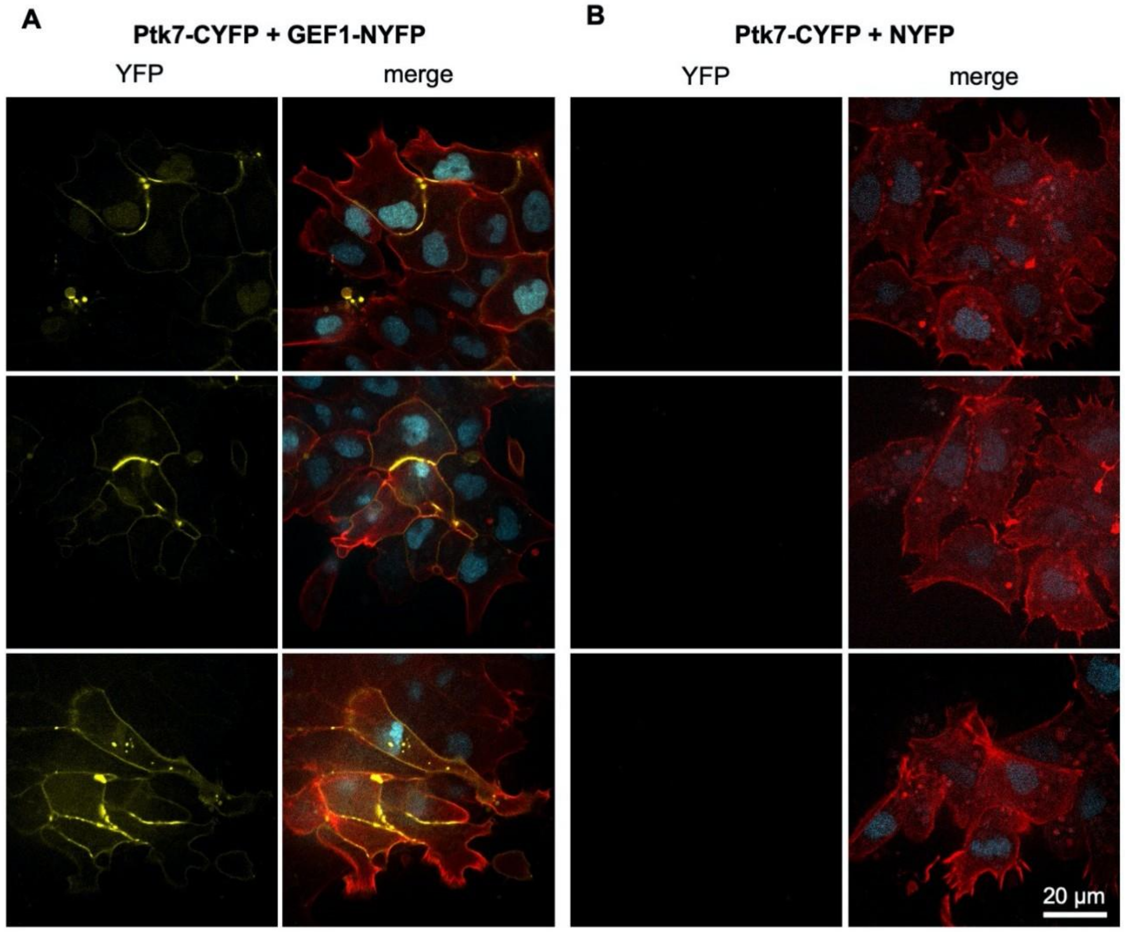

Supplementary Fig. S4: PTK7-CYFP complements with GEF1-NYFP, but not NYFP alone. NC explants were injected with 100 pg *Ptk7-CYFP* RNA in combination with 100 pg *GEF1- NYFP* RNA or 100 pg *NYFP* RNA together with 200 pg *H2B-CFP* RNA and 300 pg *lifeact-RFP* RNA in one blastomere at the two-cell stage. For each combination three representative images are shown. YFP signal indicates an interaction of Ptk7 and GEF1 at NC cell-cell contacts, while no signal is detected in explants expressing Ptk7-CYFP and NYFP using identical imaging parameters. Scale bar: 20 µm.

4. Some YFP complementation (Fig. 2D) and Rho activation (Fig. 5) signals are observed outside cell-cell contacts. It would help to see images with larger fields of view (15-20 cells each).

For the reviewer's convenience, we present here images with a larger field of view (**Fig. R2**), although we are not sure which signals the reviewer is referring to. As we know that Ptk7 is not exclusively localized at the membrane, its interaction with Trio may not be limited to the plasma membrane.

NOTE: We have removed unpublished data that had been provided for the referees in confidence.

During NC cell-cell contact formation, PTK7 accumulates at the contact zone, but is removed by endocytosis when these cell-cell contacts are broken. During this process, Ptk7 is also detected in close proximity to the membrane and in cytoplasm (Berger et al., 2017, Grund et al., 2021). Furthermore, NC cells form cytonemes that connect NC cells.

NOTE: We have removed unpublished data that had been provided for the referees in confidence.

© 2025. Published by The Company of Biologists under the terms of the Creative Commons Attribution License (https://creativecommons.org/licenses/by/4.0/).

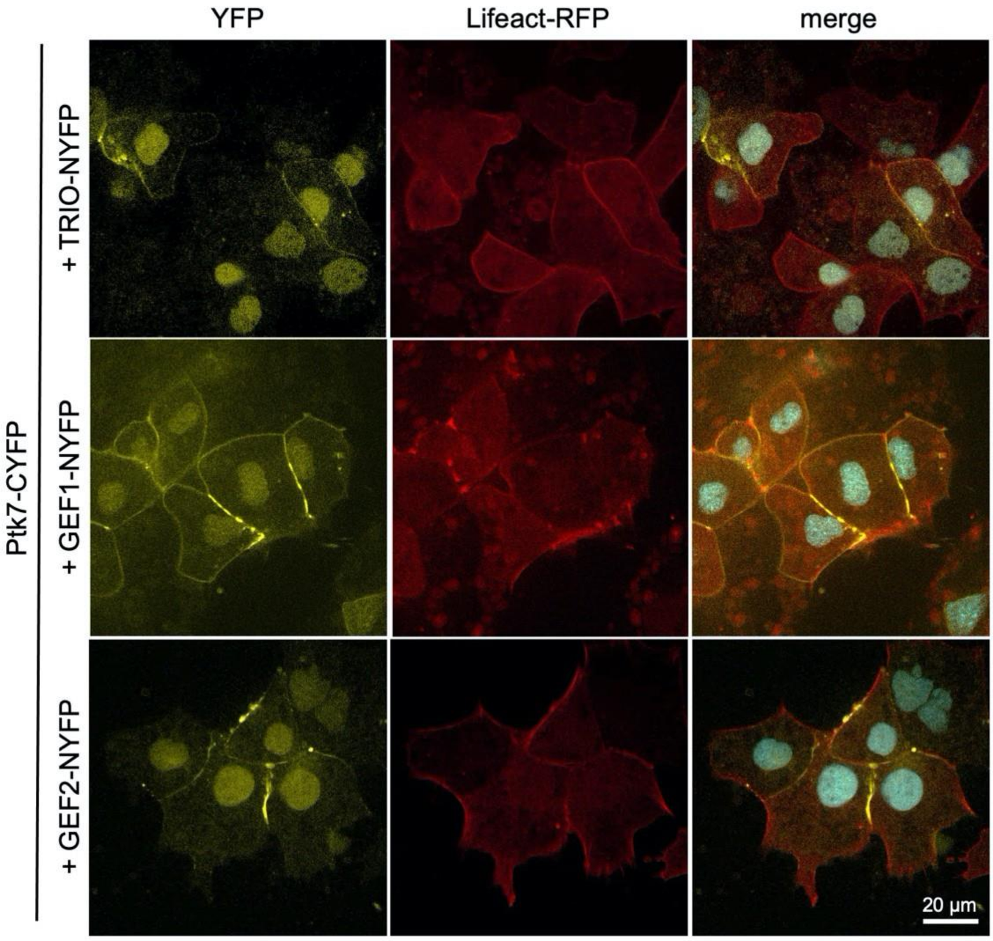

Fig. R2: Larger view of the images shown in Fig. 3A.

5. In Fig. 2A, all tested PTK7 constructs are detected in Trio-HA pulldowns, leading to the proposal that PTK7 juxtamembrane domain is critical for the binding. A direct test of this idea after the deletion of the putative interaction domain would strengthen the paper, or alternative explanations can be offered, e. g. multiple interacting domains.

We agree with the reviewer. Ideally, we could identify a single responsible domain, delete it and then show that we abolish interaction with Trio when we perform IPs using this deletion construct. One problem here is that Ptk7 mediates homophilic binding (Grund et al., 2021). Therefore, if we delete the juxtamembrane domain, the remaining protein would still be able to dimerize with endogenous Ptk7 and this dimer may still be able to recruit Trio. The situation may be even more complex, if the interaction is mediated by additional proteins that can interact with Trio (reviewed in Schmidt and Debant, 2014, van Rijssel and van Buul, 2012; Kempers et al., 2021).

We did not dedicate a larger section of the discussion to this aspect, but added a sentence (line 403-404) simply stating: „ We currently do not know if this interaction is direct, involves multiple protein domains or even additional interaction partners."

6. The Trio band in the HA pulldown is faint, raising concerns about assay specificity, as a similar band appears in the control (Fig. 2A, lanes 3 and 4), and Trio-HA expression in lysates is not shown. Can this be improved?

We have replaced the blot with one that shows the Trio band more clearly (Fig. 2A). Trio-HA expression in lysates has now been added (Fig. 2A) as well as PTK7-Myc expression in lysates (Fig. 2C).

© 2025. Published by The Company of Biologists under the terms of the Creative Commons Attribution License (https://creativecommons.org/licenses/by/4.0/).

7. Fig. 3 aims to show that Trio is required for the "contact inhibition of locomotion". On the other hand, the invasion was not affected in morphant cells. Please comment on whether Trio depletion affects the frequency of cell-cell collisions, the time of cell-cell contact and the percent of the cell perimeter involved in the contact. How was the initial velocity vector (red arrow) defined? Given the complexity of these assays, the authors should acknowledge that more than one interpretation of their results is possible.

Depletion of Trio does not significantly affect the frequency of cell-cell collisions, the duration of the cell-cell contact or the percentage of the cell perimeter involved in the contact compared to controls (**Fig. R3**). For these analysis we used exactly the same cells that we analyzed in the cell-cell collision assays shown in Fig. 4A,B, which are single migrating cells that showed no interaction 10 minutes before and after cell-cell-collision. Therefore, we cannot say how these parameters are in general affected in Trio morphants, which could be dependent of many factors like the density of cells, their circularity, ability of cells to separate etc.

Concerning the cell-cell collision assay and the definition of the initial velocity vector (**red arrow**) we have added the following information in the method section (line 169-172): „The initial velocity vector was defined and its orientation standardized as follows: the starting point describes the initial position of the cell at the beginning of the analysis (-Δt), end point marks the position of the cell at the time of collision (t=0). Black arrows indicate the direction of migration after collision."

We are aware that these assays are complex and difficult to interpret. We used the discussion to develop a working model, however, we tried to word this cautiously to acknowledge that there may be other explanations.

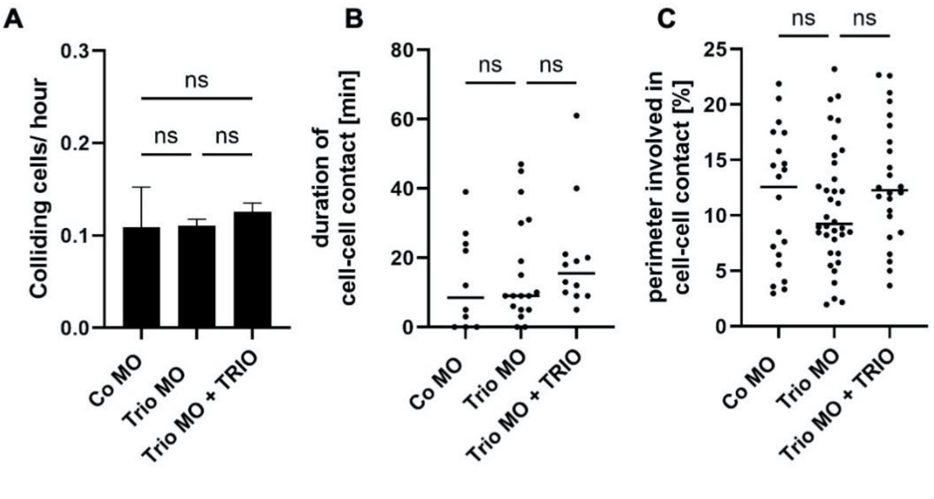

Fig. R3: Single cell migration parameters that are unaffected by Trio depletion. The graphs represent the frequency of cell-cell collisions **(A)**, the duration of cell-cell contact **(B)**, and the percentage of the cell perimeter involved in the contact **(C)** for the collision events analyzed in Figure 4A-C. Number (*n*) of analyzed NC cells of three independent experiments: Co MO = 20, Trio MO = 36, Trio MO + TRIO = 24. **(A)** Data are mean ±s.e.m., ns = not significant (one-way ANOVA). **(B)** The duration of cell-cell contact was determined by measuring the time between the first frame in which the cell-cell contact was observed and the last frame showing contact. Since multiple positions were recorded rather than a continuous movie of each collision, cells that appeared in contact in only a single frame were assigned a duration of 0 min. The median is plotted as a line, ns = not significant (Kruskal- Wallis test). **(C)** To determine the percentage of the cell perimeter involved in contact, the frame representing the middle point of the contact phase was selected. The cell outline was measured, and the percentage of the perimeter in contact with another cell was calculated. The median is plotted as a line, ns = not significant (Kruskal-Wallis test).

© 2025. Published by The Company of Biologists under the terms of the Creative Commons Attribution License (https://creativecommons.org/licenses/by/4.0/).

8. The partial rescue of neural crest migration of PTK7 morphants by caRhoA, caRac1 and caCdc42 (Fig. 4) seems at odds with the rescue shown with dnRac (Fig. 6) and deserves a comment.

There is an improvement of the Trio morphant phenotype by co-injection of caRac1 and caRhoA, but only the rescue effect with caRhoA is significant. Furthermore, co-injection of caCdc42 does also not rescue. The question is why do caRhoA and dnRac1 both rescue? While we think that the dnRac1 compensates for the blockage of the GEF1 domain by Ptk7, the RhoA effect may be indirect. Ptk7 loss of functions leads to a blebbing phenotype and a loss of cell protrusions, it is possible that RhoA may simply counteract this. For example, in the zebrafish lateral line primordium pulses of RhoA signaling stimulate actin polymerization and flow in protrusions to drive collective cell migration (Qian, 2023). Furthermore, since the small GTPases of the Rho family have the ability to bidirectionally affect their activity (Burridge and Wennerberg, 2004), RhoA activation may also indirectly affect the activity of Rac1.

9. Does the association of PTK7 with Trio affect the binding of Trio to other targets that modulate NCC migration, including Dvl, Cadherin 11 or Par3? While it is not directly relevant, this knowledge may provide further support to the proposed mechanism.

This is an interesting question that we have also been thinking about, but which is difficult to address. We have started to look into the interaction of Trio and Dvl and analyzed whether this is affected by Ptk7 loss-of-function. Using co-immunoprecipitation experiments, it turns out to be very difficult to quantify these effects, as binding efficiencies vary between experiments/embryo batches making it difficult to distinguish real effects from experimental variation. Therefore, this will be a question that remains to be answered in the future.

Response to Reviewer 2

1. Localized vs. Global Rac1 Inhibition:

The model proposed by the authors suggests a localized regulation of RhoA and Rac at the cell contact, consistent with previous studies (e.g., Carmona-Fontaine et al., 2008). However, in Fig. 6, they demonstrate that inhibition of Ptk7 is rescued by global (not localized) inhibition of Rac1. How is this possible? How does the inhibition of Rac1 throughout the cell, including protrusions known to be Rac1- dependent, not impair neural crest migration? A potential experiment to address this question would be to analyze cell protrusions using the neural crest explant assays shown in other figures. Perhaps cell protrusions are not completely inhibited in the dnRac1, and this residual activity is sufficient to drive directional migration in the embryo.

We shared the concerns of the reviewer and have only used concentrations of dnRac1 at which we do not see developmental defects. At these concentrations, we also do not observe major defects in NC protrusion formation and migration. To show this, we performed the experiment that was suggested by the reviewer (**Fig. R4**). Embryos were injected with 10 pg dnRac1 DNA and NC explants were analyzed by immunostaining using the Rac1-GTP antibody. There is still residual Rac1 activity in protrusions although reduced compared to controls. The cells are still able to form protrusions and the cell circularity is comparable to wild-type cells.

© 2025. Published by The Company of Biologists under the terms of the Creative Commons Attribution License (https://creativecommons.org/licenses/by/4.0/).

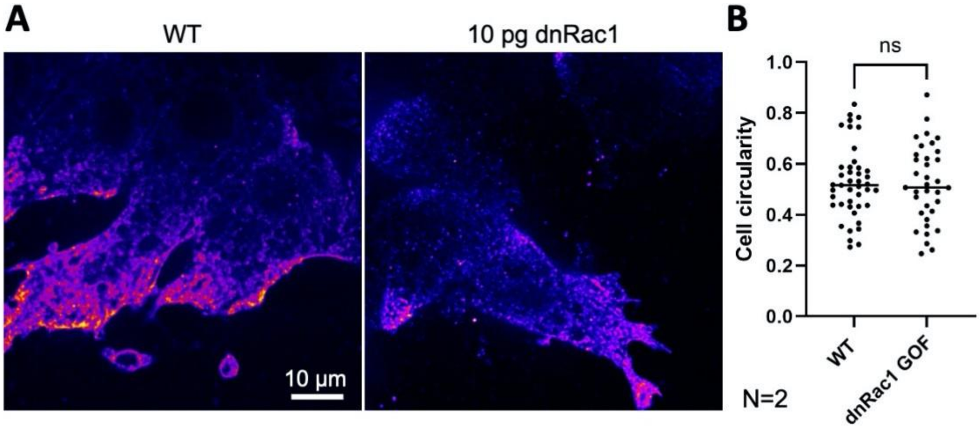

Fig. R4 Expression of dnRac1 does not affect protrusion formation of NC cells.

Concerning the question of the reviewer, how dnRac1 can compensate for loss of Ptk7, without affecting NC migration, we speculate that the increased Rac1 activity at cell-cell contacts in Ptk7 morphants is still significantly lower than the high Rac1 activity at cell protrusions. Therefore, in a NC cell expressing global dnRac1 this decrease in Rac1 activity may be more notable at the cell-cell contact than in the NC cells protrusions.

2. Twist Expression and Neural Crest Migration:
***Some experiments described as showing inhibition of neural crest migration also report reduced expression of Twist in neural crest cells. How do the authors rule out the possibility that impaired migration is a consequence of a reduced number of neural crest cells (fewer cells expressing Twist) due to an early effect on neural crest formation or induction? To address this, the authors could examine Twist expression, or another neural crest marker, at earlier (premigratory) stages**.*

As suggested by the reviewer, we analyzed whether loss of Ptk7 function affects NC induction. We injected the same concentration of MO that we used for the NC migration assay and analyzed any changes in the *twist* expression pattern at NC induction stages and did not detect any significant defects. These data have now been included as supplementary **Fig. S1**.

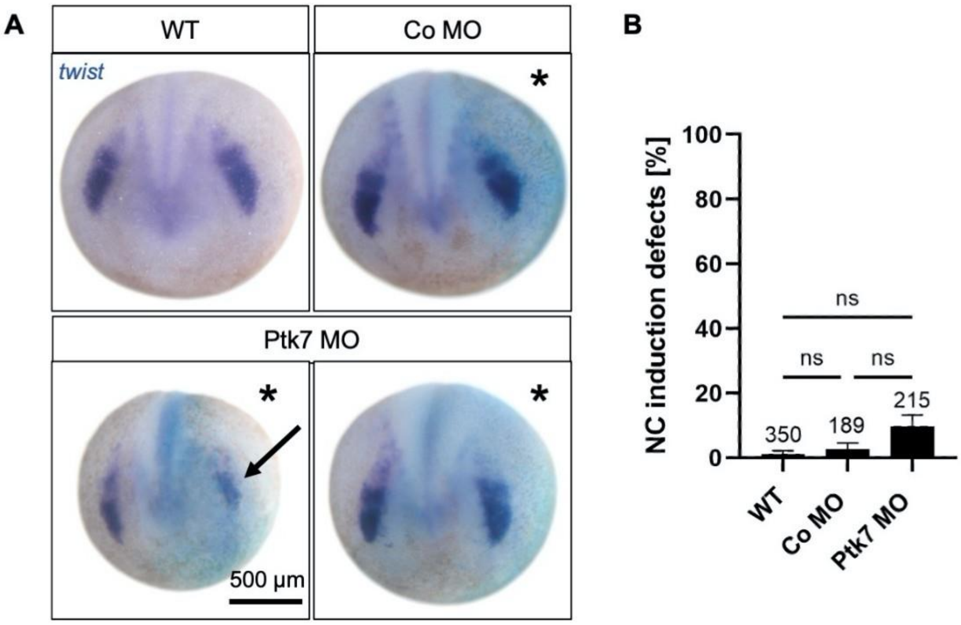

© 2025. Published by The Company of Biologists under the terms of the Creative Commons Attribution License (https://creativecommons.org/licenses/by/4.0/).

Supplementary Figure S1: Ptk7 loss-of-function does not affect NC induction. Two-cell stage embryos were injected with 7.5 ng Ptk7 MO or a control MO in combination with 75 pg *lacZ* RNA as lineage tracer. **(A)** NC cell induction was analyzed at stage 17/18 by *twist in situ* hybridization. Asterisks mark the injected side. Arrow indicates NC induction defect. Scale bar: 500 µm. **(B)** Graph showing NC induction defects of four independent experiments. The total number of embryos is indicated for each column. Data are mean ±s.e.m., ns = not significant (one-way ANOVA).

3. Bimolecular Fluorescent Complementation Controls:
The bimolecular fluorescent complementation assay effectively demonstrates the interaction between Ptk7 and Trio. However, the controls (with the individual molecules), which are currently presented in the supplementary figure, should be included in the main figure. These controls are critical for correctly interpreting the data.

We thank the reviewer for this comment and have added the data in the **new Fig. 3**.

4. ***Contact Inhibition of Locomotion vs. Invasion Assay:***
The authors report a clear effect of Trio inhibition on contact inhibition of locomotion, but no effect on the invasion assay (Fig. 3D). This is somewhat puzzling, as these assays are typically consistent. Since this is a negative result, including a positive control that demonstrates increased invasion after impairing contact inhibition of locomotion would strengthen the conclusion. However, I do not consider this essential as long as the data (number of experiments, statistical analysis, etc.) for Fig. 3D are robust.

Katharina Till who conducted these experiments also performed the same experiments with **Ptk7 MO injected explants** and could indeed see a **defect in cell invasion** (Grund et al., 2021, **Fig. R5**). Therefore, we were also surprised that we did not see these defects with Trio morphant NC cells. An explanation may be that Ptk7, whose extracellular domain is apparently crucial to protect cells from NC cell invasion, is still present in Trio morphant NC cells.

© 2025. Published by The Company of Biologists under the terms of the Creative Commons Attribution License (https://creativecommons.org/licenses/by/4.0/).

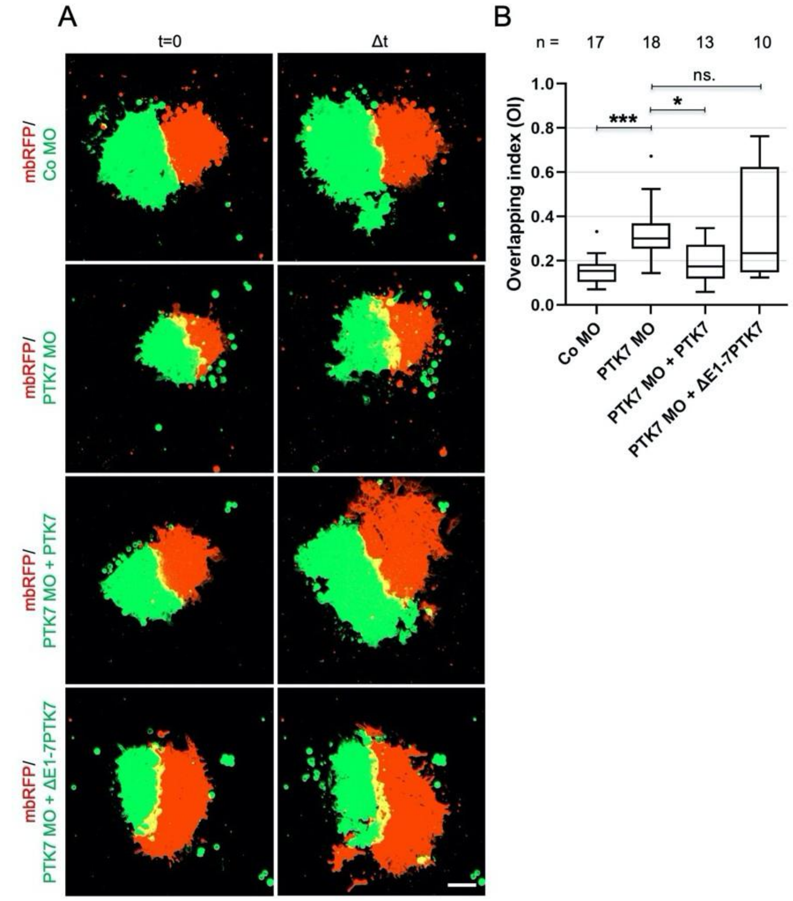

Figure R5: NC cells invade Ptk7 morphant NC cells. Confrontation assays were performed using explants injected with either 6.5 ng MO in combination with 100 pg *mbGFP* RNA or 200 pg *mbRF*P RNA as control. For rescue experiments 200 pg PTK7 or ΔE1-7PTK7 RNA were co-injected with the MO. (A) Confronted explants at the start of the experiment (time point t = 0) and at the time point of the maximal invasion (Δt). (B) Box plot showing overlapping indexes (OI) of at least three independent experiments. The median is plotted as a line inside the box. * $p < 0.05$, *** $p < 0.005$, ns.: not significant (Kruskal–Wallis test) (cited from Grund et al, 2021).

Second decision letter

MS ID#: dev.204446R1

MS Title: The Rho GEF Trio functions in contact inhibition of locomotion of neural crest cells by interacting with Ptk7

Authors: Annette Borchers; Katharina Till
Article Type: Research Article

Dear Annette,

I am happy to tell you that your manuscript has been accepted for publication in Development, pending our standard publication integrity checks.

Reviewer 1

Advance summary and potential significance to field

© 2025. Published by The Company of Biologists under the terms of the Creative Commons Attribution License (https://creativecommons.org/licenses/by/4.0/).

This paper assesses the interaction of PTK7 and Trio in neural crest migration in Xenopus development. Pull downs from transfected HEK293T cell lysates and YFP complementation assays in embryonic cells demonstrate the association of PTK7 with Trio at cell-cell contacts. Since Trio activates Rac and Rho through two distinct GEF domains, the authors propose a model, in which PTK7 suppresses Rac1 activity and favors the RhoA GEF activity at cell junctions.

Comments for the author

The authors satisfactorily addressed the majority of my comments. The paper has been sufficiently improved for publication to be recommended.
Please include Figure R3 data in the revision, these results would be useful.

Minor comments: line 126: "...likely functions downstream or in parallel with..."
Line 154: it appears as if the interaction has been tested in NC cells. Please indicate that it was done in HEK293 cells.

Reviewer 2

authors have adequately address my previous concerns

© 2025. Published by The Company of Biologists under the terms of the Creative Commons Attribution License (https://creativecommons.org/licenses/by/4.0/).
